# High-Throughput Sequencing and Mutagenesis to Accelerate the Domestication of *Microlaena stipoides* as a New Food Crop

**DOI:** 10.1371/journal.pone.0082641

**Published:** 2013-12-18

**Authors:** Frances M. Shapter, Michael Cross, Gary Ablett, Sylvia Malory, Ian H. Chivers, Graham J. King, Robert J. Henry

**Affiliations:** 1 Southern Cross Plant Science, Southern Cross University, Lismore, New South Wales, Australia; 2 Native Seeds Pty Ltd, Sandringham, Victoria, Australia; 3 Queensland Alliance for Agriculture and Food Innovation, University of Queensland, Brisbane, Queensland, Australia; Temasek Life Sciences Laboratory, Singapore

## Abstract

Global food demand, climatic variability and reduced land availability are driving the need for domestication of new crop species. The accelerated domestication of a rice-like Australian dryland polyploid grass, *Microlaena stipoides* (Poaceae), was targeted using chemical mutagenesis in conjunction with high throughput sequencing of genes for key domestication traits. While *M. stipoides* has previously been identified as having potential as a new grain crop for human consumption, only a limited understanding of its genetic diversity and breeding system was available to aid the domestication process. Next generation sequencing of deeply-pooled target amplicons estimated allelic diversity of a selected base population at 14.3 SNP/Mb and identified novel, putatively mutation-induced polymorphisms at about 2.4 mutations/Mb. A 97% lethal dose (LD_97_) of ethyl methanesulfonate treatment was applied without inducing sterility in this polyploid species. Forward and reverse genetic screens identified beneficial alleles for the domestication trait, seed-shattering. Unique phenotypes observed in the M2 population suggest the potential for rapid accumulation of beneficial traits without recourse to a traditional cross-breeding strategy. This approach may be applicable to other wild species, unlocking their potential as new food, fibre and fuel crops.

## Introduction

Cereal production is the major source of carbohydrate in human diets, with most provided by eight genera of the Poaceae [1]. Increasing world population, climate variability and reduced agricultural land, water and associated inputs drive a need to develop new food crops. New cereal species, able to be cultivated in more marginal environments should be considered. Wild grass species intrinsically adapted to marginal environments and climatic variability, provide an excellent target for domestication.

Australia is a unique source of under-utilised germplasm, due to its short agricultural history, geographic isolation and relative lack of arable land, plant domestication. The Australian Poaceae have evolved independently of other world environments. Moreover, they are adapted to a wide range of resource-limited environments, providing a novel genetic resource for plant pre-breeding. To date, no commercial cereal crops have been developed from this gene pool, although there has been a history of traditional use by indigenous Australians [2].

**Figure 1:**
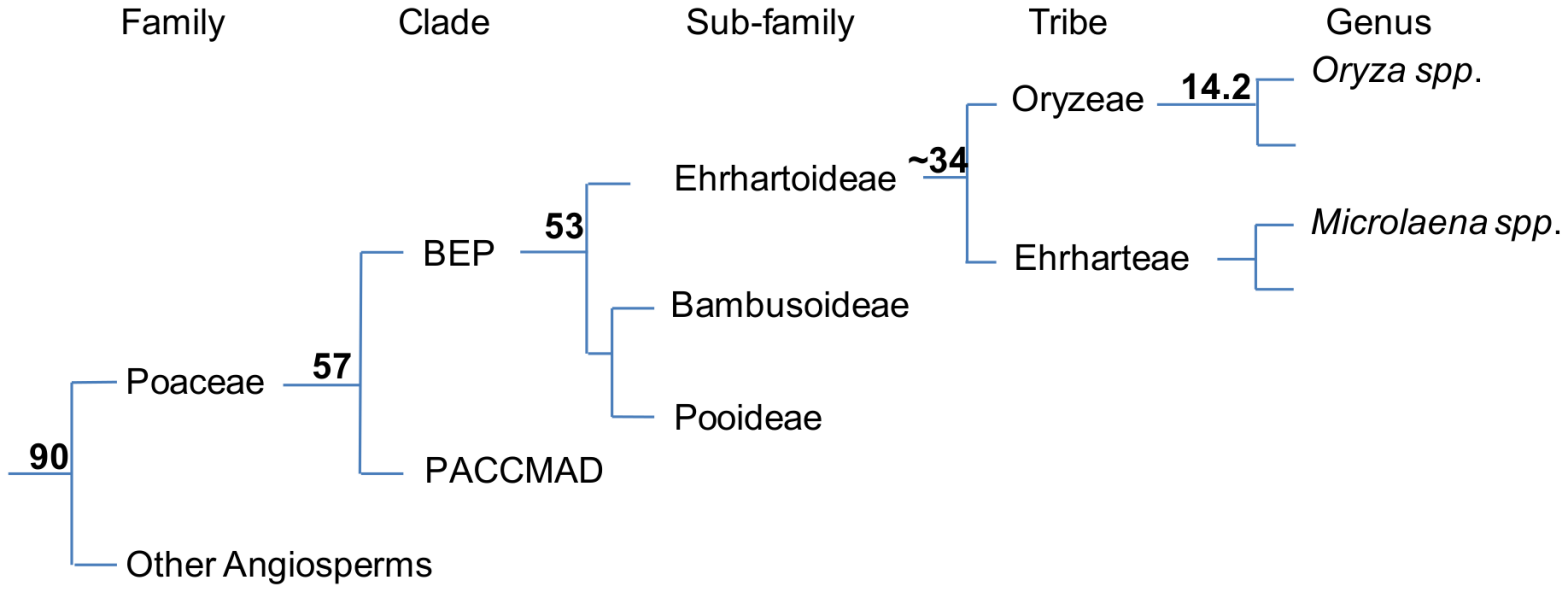
Composite figure outlining the evolutionary relationship between *Microlaena stipoides* and cultivated rice (*Oryza sativa*). Nodal numbers reflect the estimated million years since each bifurcation [4]–[6]. Although *O. sativa* and *M. stipoides* shared their last common ancestor approximately 34 million years ago they retain the same base chromosome number, n = 12, similar individual genome sizes [3], endosperm morphology and characteristics [8] and genetic homology [7].

Approximately 35 million years ago *Microlaena stipoides* (weeping rice grass) shared a common ancestor with cultivated rice, *Oryza sativa* (Figure 1; [3]–[8]). *M. stipoides* was one of the first Australian grasses identified as having potential to be domesticated as a new cereal crop [9]. Its large grain size, plant architecture, suite of adaptations to marginal and variable environments, and high level of intra-species diversity have been widely recognised [10], [11]. Additionally, *M.stipoides* has the same base chromosome number as rice (n = 12) and its tetraploid genome size (880 Mbp) is approximately double that of diploid *O. sativa* (394 Mbp) [3], [12].

Increased availability of genomic data has contributed to a better understanding of the genetic basis of the so-called “domestication syndrome” in commercially cultivated Poaceae [13]–[16]. Indeed, for many domestication traits such as seed shedding upon ripening (shattering), grain color, awn length, dwarfing, grain size, grain number and panicle shape, quantitative trait loci and gene sequences have been identified [17], [18]. Although domestication traits may often be controlled by a network of genes, the loss of function of a single component may result in phenotypic modification. For example, seed shattering in rice may be eliminated due to a loss of function of either *qSH1* [19] or *sh4* [20]/SHA1 [21]. Loss of function may result from single base polymorphisms, either naturally occurring or induced [22]. Therefore in most cases the domesticated phenotype results from the cumulative loss of function of multiple genes.

By 2004, mutant-derived *O. sativa* lines had an estimated global value of over US$20 billion [23]. Ethyl methanesulfonate (EMS), a water soluble mutagen alkylates the DNA nucleobase, guanine. This results in randomly distributed point mutations throughout the genome, which in the majority of cases are GC→AT transitions [24]. Targeting Induced Local Lesions IN Genomes (TILLING) has enabled reverse genetic screening of EMS-mutagenised populations [24]–[27], with several recent modifications to the protocol [28], [29]. More recently, Next Generation Sequencing (NGS) using ‘short read’ platforms such as the Illumina GAII provide a cost effective and informative option for reverse genetic screening of large mutant populations [30], [31].

We aimed to accelerate the process of crop domestication by identifying variation in specific traits and their underlying component genes. We harnessed the sensitivity of NGS to characterise both natural and EMS-induced variation within bulked amplicon pools, identifying candidate alleles for improved breeding of the semi-domesticated species, *M. stipoides*.

## Methods

### Plant material and EMS treatment protocol

A seventh generation, predominantly inbred, breeding line of *M. stipoides*, cv AR1, was supplied by Native Seeds Pty Ltd (nativeseeds.com.au, last accessed 27/7/11) as our base material. Due to poor germination rates in *M. stipoides* when dehusked, seeds where treated with husks intact and all florets included in the trial were manually checked to ensure the husk contained a filled seed. Imbibition was conducted at a concentration of approximately 10 g of seed per 40 ml of solution (adapted from [32]). Seeds were pre-soaked in water for six hours, then imbibed in either de-ionised water, 40, 60, 80, 100, 115, 130, 145, 160, 175 or 200 mM aqueous solutions of ethyl methanesulfonate (EMS) for 18 hours on a Bio-line orbital shaker at 160 cycles/minute at 22 degrees centigrade in 200 ml Schott bottles. The treatment solution was decanted and 40 mL of de-ionised water was added as a wash solution. This was repeated every 15 mins for four hours. Seed was then immediately planted into germination trays containing Searle's premium potting mix (http://www.searle.com.au/PottingMixes.html, last accessed 01/10/13) and grown under glass house conditions with average minimum and maximum daily temperatures of 15°C and 24°C respectively.

### Optimisation of EMS treatment for *M. stipoides*

**Figure 2:**
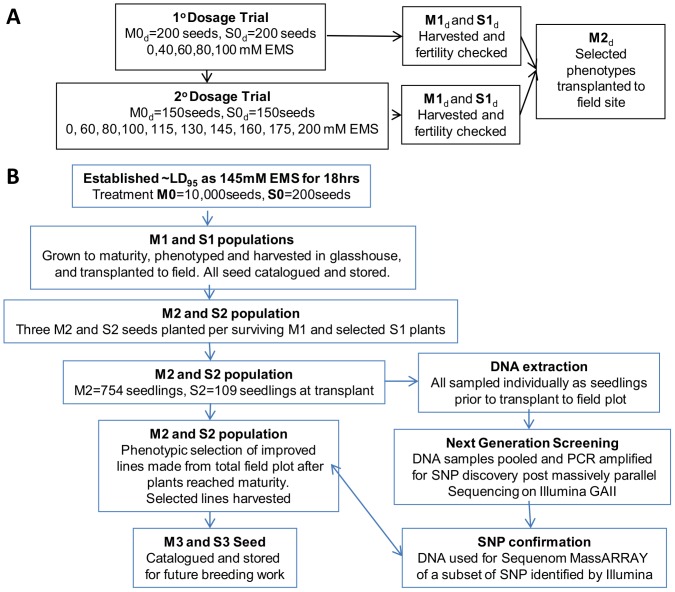
An overview of experimental flow identifying initial optimisation of appropriate EMS treatment (A) and development of the control and mutant populations for reverse genetic screening for SNP discovery and validation. Note, mutant generational nomenclature is as per McCallum et al 2000, where _d_ denotes dosage trial, M indicates EMS treated seed and S indicates the selfed control line.

Based on effective doses used in other Poaceae, an initial dose response curve with a control and four treatments of 40, 60, 80 and 100 mM EMS (200 seeds/treatment) was generated. A second dose response experiment was then conducted to ascertain the efficacy endpoints for *M. stipoides* with a control and nine treatments of 60, 80, 100, 115,130,145,160,175 and 200 mM EMS (150 seeds/treatment). Germination was monitored and recorded every 2–3 days for a month for the mutated, M1_d_, and control seedlings, S1 _d_, (Figure 2; generational nomenclature as per [25], where _d_ denotes dosage trial, M – EMS treated and S- selfed control plants). In order to maximise mutation density in the polyploid background, an LD_95_ was targeted with final percentage germination calculated at 6 weeks post treatment resulting in an LD_97_ being achieved. Seedlings were then monitored on an ongoing basis for any notable phenotypic variations. Seedlings were transplanted into individual 6 cm×6 cm×15 cm forestry tubes of Searle's premium potting mix and grown to maturity on a semi shaded roof top with water applied as required to keep the potting mix moist. M2_d_ and S2 _d_ seed was collected individually from all non-sterile mutants. Due to the perennial habit of *M. stipoides* both M1 _d_ and M2_d_ individuals from the dosage trial which displayed a promising or novel phenotype could be retained and were transplanted to a field site for final evaluation.

### Development of the EMS mutant breeding population

For the 145 mM EMS screening population, both the control (200) and treatment (10,000) seeds were screened for grain fill prior to the water or 145 mM EMS treatment. *M. stipoides* has a predominantly cleistogamous (selfing) reproductive system, though it can also exhibit opportunistic chasmogamous (outcrossing) breeding cycles [33]. The latter was rarely observed and always recorded during the experiment. The M1 population and its control plants (S1) were evaluated under glass house conditions until maturity when M2 and S2 seed was harvested with novel phenotypes recorded at harvest. M1 and S1 plants were then trimmed and transplanted into the field site for phenotypic observation. Three M2 and S2 control seeds per M1 or S1 plant were planted and subsequent germination percentages recorded. Leaf tissue was collected from all individuals in the 145 mM M2 and S2 population followed by transplantation into the field site. At transplantation to the field site all mature plants were trimmed to approximately 5 cm above the culm. This decreased the stress on the plant and reduced the phenotypic variability resulting from the glasshouse environment.

### Illumina sequencing of pooled amplicons

The M2 and S2 populations were used as the basis for genotypic screening. Leaves were collected from 754 juvenile M2 seedlings and 109 S2 individuals. DNA was extracted from fresh leaf tissue using a modified MagAttract 96 DNA Plant Protocol (Qiagen, Frankfurt, Germany) with one additional reverse osmosis purified water wash prior to being quantified using UV spectrophotometry at a wavelength of 260 nm and 280 nm (MWG Sirius Plate reader, MWG Biotech, Ebersberg, Germany). The DNA was normalised using Gibco Nuclease Free water to a concentration of 2 ng/µl using the MWG Theonyx (MWG Biotech, Ebersberg, Germany). Prior to amplification DNA was pooled from five individuals and 10 ng of pooled template was used per PCR. Stringent quality controls were applied during sample preparation. DNA was quantified, normalised and pooled in equimolar proportions at each step in an attempt to maintain relative allele frequencies in the subsequent GAII sequence data.

Four candidate domestication related homologues were targeted for PCR amplification (Table S1 in File S1). Homologues of granule bound starch synthase 1 (GBSS1), encoded by the *Waxy* gene [7], the *Isa* gene [34] and two gene homologues controlling seed shattering in rice, *sh4*/*SHA1* and *qSH1* [35] identified in *M.stipoides* were targeted. PCR products were quantified by gel electrophoresis using Scion image (http://softwaretopic.informer.com/scion-image-free-software/, last accessed 01/10/13). Amplification products were combined in equimolar amounts to form homologue-specific pools of 109 and 754 M2 (mutant) individuals, in addition to a pool of 109 S2 (control) individuals. The homologue-specific pools were then quantified by pico-green and combined in equimolar amounts to form megapools representing two mutant and one control population. These three megapools were run as individual lanes on the Illumina GAIIx platform (Illumina, San Diego, CA, USA) using a paired-end strategy with a fragment size of 400 bp and a read length of 75 bp.

Sequence data were trimmed using CLC Genomics Workbench version 4.0.3 (www.clcbio.com, last accessed 02/010/13). Reads with a quality score of less than 0.001 were discarded and paired-end reads were trimmed to a minimum of 30 bp. Reference assembly against *M. stipoides* sequence (Genbank accessions; EF600044, HQ008270, HQ008271, HQ008272) was undertaken with a mismatch cost of 2, insertion and deletion costs of 3, length fraction of 0.8 and similarity of 0.8, minimum distance for paired end reads of 180 bp with a maximum of 340 bp, and non-specific matches ignored. SNP detection parameters of; window length 21, maximum number of gaps or mismatches 2, SNP minimum quality score 30 and quality score for the surrounding bases 30, minimum coverage required 1×, with a minimum variant frequency of 0.000001%, was designed to capture all high read quality polymorphisms.

### SNP discovery

Analysis of the CLC SNP discovery output was conducted using Microsoft Excel 2007 following parameters in line with the currently reported error limitations of the Illumina GAII platform for pooled rare SNP discovery [30]. A minimum coverage requirement was set at 400× (approximately 10× the effective pool size for the 109 pools). Based on alignment of these gene homologues and their splice junction sites to rice, putative exon/intron boundaries were assigned to the *M. stipoides* reference sequence [7] and this was used to assign putative functionality of the SNPs.

We carried out an assessment of site-specific variability by calculating the information-content at each nucleotide position [36], to test an error threshhold of 0.5% for these data. The work of Tsai et al 2011 indicated that SNP calls with a frequency >0.5% are unlikely to be false positives and that in all cases the predicted frequency from the Illumina GAII data will be higher than a SNPs actual or theoretical frequency, due to the addition of erroneous ‘noise’ inherent at all reference positions in Illumina GAII data.

### Sequenom MassARRAY SNP confirmation

A subset of 24 SNPs of interest was incorporated into a Sequenom SNP assay. PCR and single base extension primers for each SNP investigated were designed using Assay Design software, version 4.0 (Sequenom Inc., San Diego, CA). The genotyping was performed according to the iPLEX Gold SNP protocol on the Sequenom MassARRAY Compact platform and analysed using Typer 4.0.

## Results

### Determination of optimal dose of EMS

We established an optimal EMS treatment for *M. stipoides* using a two stage experiment based on final germination frequency (Figure S1). Figure 2 provides an overview of the experimental methodology. The closest approximate to a LD_95_ dose determined was a 145 mM EMS treatment, which resulted in 3% germination (LD_97_). To ensure that sterility had not been induced [38] seed from plants within both the M1_d_ and S1_d_ (where M# (mutant) and S# (selfed control) refer to the generation and _d_ denotes the dosage trial) populations were harvested. Germination tests confirmed the viability of the M2 seed at this dose.

### Phenotypic analysis of the mutant population

**Figure 3:**
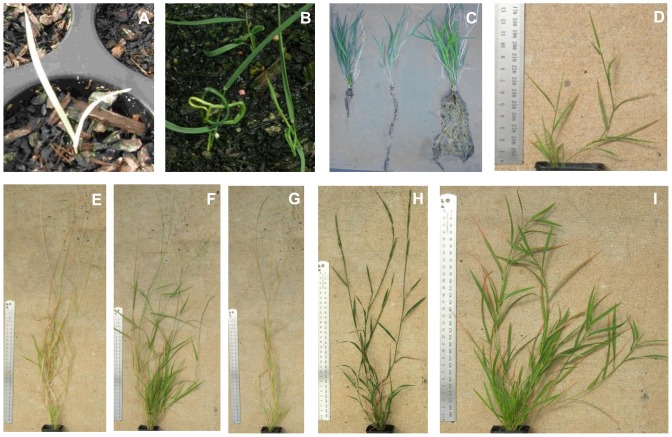
Examples of mutant plant phenotypes with differing doses of EMS and developmental stages. **A**. 145 mM EMS treated chlorophyll aberration, only observed in M2 145 mM population, and not observed across all sibling M2 seedlings **B**. 130 mM treated mutant seedling **C**. 115 mM EMS treated mature plants showing root variation within pot trial **D**. 145 mM treated dwarf, no seed produced **E**. control **F–I**. 145 mM mature plants showing mutant phenotypes not seen in control populations; variations to plant architecture, leaf width, length and color, plant vigor, panicle shape, seed production and synchrony of maturity, and inter-nodal span length **I**. Individuals with this plant architecture did not produce seed. Other novel phenotypes observed in the mutant population included rhizome production, crooked nodes, non-surviving dwarfs, and sectoring as variegated leaves.

Survival at 42 days post treatment was 9% in the mutant population (10,000 seeds), compared with 82.5% in the control population (200 seeds). Novel phenotypic variations amongst the surviving mutant seedlings were observed throughout development (Figure 3). At harvest two M1 individuals underwent anthesis, with the remaining population exhibiting the typical cleistogamous breeding cycle. Seed was harvested from multiple tillers per plant. Overall M2 seedling survival was 82% compared with 91% in the S2 control line. Chlorophyll aberrations were observed in the M2 seedlings at low frequency (1.2%), but were absent in the S2 control population.

**Figure 4:**
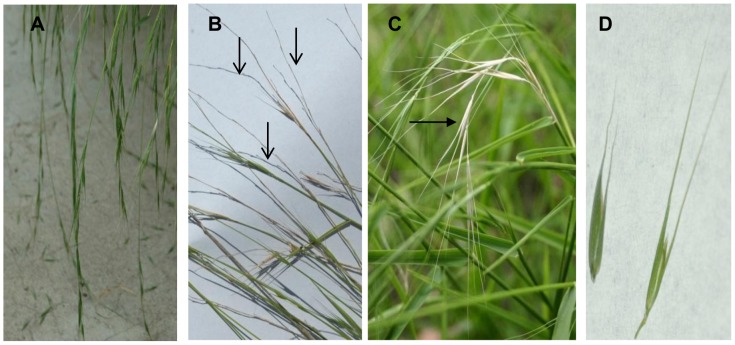
Panicle shattering habit and awn length variations observed in *Microlaena stipoides*. **A**. wild-type shattering habit with individual grains dehiscing as they reach maturity, and lodging seed heads **B**. Typical wild-type seed head showing empty panicle (↓) by the time the lower seeds have reached maturity **C**. Non-shattering panicle with all seeds retained at maturity (→) **D**. Short versus long awned grains. Short awned varieties are highly desirable as they minimise difficulties associated with handling, processing and mechanisation of the production system.

Field based evaluation of M2 mutants identified 50 plants (Table 1) with component traits contributing to a more ‘domesticated’ phenotype than the original base material. These component traits included higher grain yield, plant dry matter, erect seed head architecture, reduced- or non-shattering seed heads and larger grain size. Twenty four M2 plants possessed our primary domestication target, the non-shattering phenotype (Figure 4). M3 seed and phenotypic data were collected and are currently being evaluated in growth trials, as a grain crop for human consumption.

**Table 1:** Total *Microlaena stipoides* population numbers and treatment distribution in final field site compared with top 50 mutant phenotypes selected for their amenability to domestication and the component phenotypes observed in these groups.

Treatment	Number transplanted to field	Number of mutants selected with improved phenotypic traits	Phenotypic characteristics selected for, because of their role in accelerating the domestication process
S1 control	148		
S2 control	174		
M1 145 mM	462	11	HTN, EH, NS, HH, DS, BL, CP, LM, TT,
			LL, EP, LM, CSH, LG
M2 40 mM	90	1	EH, HH
M2 60 mM	135	7	HTN, EH, NS, HH, LG, HY
M2 80 mM	105	1	CP, EH, LG, NS,
M2 100 mM	100	1	NS, EH, HH, HTN, BU
M2 115 mM	43	2	VHH, EH, HTN, ST, CSH
M2 130 mM	29	3	HTN, EH, NS, HH, FO, HY
M2 145 mM	872	23	HTN, EH, NS, HH, DS, BL, CP, LM, TT,
			LL, EP, LM, CSH, LG, CL, PA
M2 175 mM	4	2	CP, HTN, NS, HH
Total	2162	50	

**Legend:** BL-broad leaves, BU-blue green leaf color, CP-compact plant, CSH-compact seed head, DS-delayed shattering, EH-erect habit, FO-forage application, HH-high herbage (for grazing applications), HTN-high tiller number, LG-large grains, LL-low leaf material (for grain applications), LM -late maturing, NS-Non-shattering, PA-purple awn, TT-thick tillers, V-very. **Note:** All 145 mM treated plants were progressed to the final evaluation while the S1/S2 controls and M1/M2 dosage trial populations underwent selection for potentially beneficial phenotypes, particularly reduced shattering, prior to transplant.

### Next generation sequencing (NGS) for SNP discovery

The four target genes, (*isa, qSH1, sh4* and *waxy*) were selected for their impact on domestication in other cereals and sequence availability in *M. stipoides*. Preliminary Sanger sequencing (data not shown) of wild type individuals identified within-individual polymorphisms assumed to result from multi-locus variation due to either tetraploidy, and/or heterozygosity. In addition, SNP variation was found between individuals in the base population suggesting a degree of out-crossing.

A PCR pooling strategy was used for NGS analysis, creating cost effective, single lane experiments characterising SNP type and frequency for each gene. Three amplicon pools (109 control plants, 109 mutant plants and a screening pool of 754 mutant plants) were sequenced on the Illumina GAII. After stringent trimming the three pools retained the following read numbers and average read lengths (ARL); Control pool (∼58 million reads, ARL – 67 bp), 109 mutants pool (∼66 million reads, ARL – 63 bp) and 754 mutants pool (∼48 million reads, ARL – 55 bp). Reads which assembled to the reference genes were submitted to the NCBI database (Bioproject ID: SRP030218, Biosample ID: SRS486800; Control_109: SRR1001453, Mutant_109: SRR1001454, Mutant_754: SRR1001455 (http://www.ncbi.nlm.nih.gov/biosample/2361099, last accessed 30/10/13)). Subsequent to reference assembly and application of a minimum coverage threshhold of 400×, an average coverage was calculated for each pool; 109 control plants - 27118×, 109 mutants -32289× and 754 mutants -10171×. Coverage was both gene and pool dependent, as previously reported for the Illumina GA platform [39]. Site specific assessment of the SNPs [36], indicated the use of a 0.5% minimum SNP frequency threshold for sequencing error (noise). This also clearly identified SNPs which were shared between pools or unique to a single pool (Figure S2).

**Figure 5:**
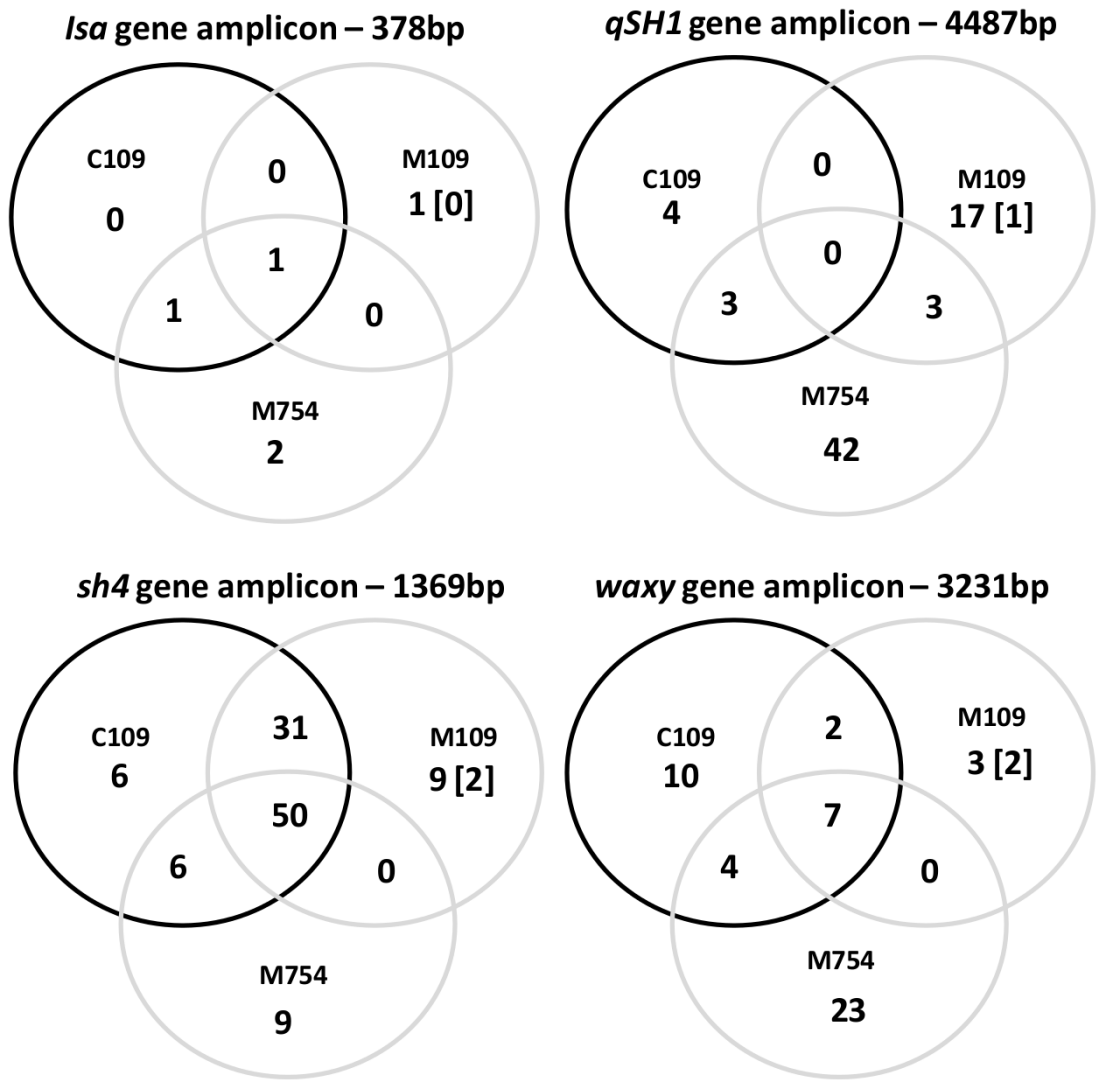
Venn diagram identifying the number of SNP identified in each of the three pools: C109 pool containing 109 wild-type individuals, M109 pool containing 109 145 mM EMS treated mutants and M754 pool containing 754 145 mM EMS treated individuals. The number of putatively EMS induced SNP (rare G/A or C/T polymorphism only found in mutant pools) is in square brackets. Full descriptions of SNPs are available in Table S2 in File S1.

NGS identified SNPs in each of the four genes examined. The shattering genes, *qSH1* (69 SNPs) and *sh4* (111 SNPs) had a greater number of SNPs identified than the *waxy* gene (49 SNPs) or the *isa* gene (5 SNPs). Each of the control/mutant pools had a distinct distribution of SNPs either unique to a single pool, or shared between multiple pools (Figure 5). In total, 234 SNPs were identified across the four target genes (Table S2 in File S1), with 229 designated as natural variation, corresponding to a wildtype SNP density in the base population of 14.3 SNP/Mb. Five putatively EMS-induced SNPs were identified as unique to the M109 pool, at the theoretical allele frequency predicted for an EMS-induced mutation (0.5%–1.5%), calculated on the assumption of 1–3 individuals in the pool having a unique homozygous G/C→A/T transition SNP per genome. This would correspond to an induced mutation density of 2.4 mutations/Mb, in addition to the polymorphism found in the wildtype population.

Of the 234 SNPs identified, 46 were predicted to cause non-synonymous amino acid changes, of which three are putative stop codons. A further 43 synonymous amino acid changes were also predicted, with the remaining 145 SNPs occurring in introns. This is based on the premise that both homeologues are potentially functional and carry the polymorphism, and that no other indels or polymorphisms have disrupted the reading frame upstream of a target SNP. Sequenom MassARRAY of selected target SNP confirmed 11 of the SNP loci. Notably, the assay confirmed a wild-type C/A SNP, predicted to cause a premature stop codon in the *sh4* shattering gene. This was confirmed for two individuals which had been noted as having a non-shattering habit.

## Discussion

Determining an effective EMS treatment to induce functional point mutations depends on the species of plant, tissue type, ploidy, and the level of mutation load sustainable without inducing lethality or sterility [26], [40].When the target species' genome contains functional redundancy (due to polyploidy, or ancient genome duplication events), a higher EMS LD can be tolerated [41]. This is supported by our study, which used a LD_97_ without inducing significant levels of sterility in the M1 population. We determined the effective dose using low numbers of seed, followed by a larger-scale generation of mutants and selection. The use of EMS as a mutagen has proven a cost and time effective method for creating new combinations of desirable phenotypic component traits in *M. stipoides*.

Where seed is treated, each genetically effective cell (GEC) will be independently mutagenised [25]. In *M. stipoides*, the GEC number is unknown. Similarly, the pattern of differentiation of each genetically effective M1 sector cannot be tracked. Although M2 populations are often constructed using only a single M2 seed from each M1 plant [27], [41], we sampled multiple seeds from each M1 plant, hence our M2 population may capture heritable traits from more than one unique reproductive sector.

The range of phenotypic variation observed allowed the selection of 50 enhanced mutant plants to be used as a pre-breeding population. The selected plants exhibited a unique composite of improvements to plant architecture, reduction of shattering at grain maturity and an increased grain size and/or yield not observed in individuals within the wild-type AR1 base material.

The *M. stipoides* AR1 base material is a semi-domesticated facultative cleisotogamous polyploid with the capacity to outcross, though this was rarely observed in this study. Cloning of another undomesticated, yet cultivated polyploid Poaceae, *Echinochloa ssp.*, identified multiple homeologues of *sh4*, with sequence polymorphism confirmed between the individual's genomic copies [42]. Similarly, in the current study we expected significant levels of wild-type polymorphism both between individuals, and between genomes within each individual.

The use of a pooled amplicon-based NGS approach was effective for detecting wild-type polymorphisms and putatively, EMS-induced mutations. However, there are limitations to using the Illumina platform for this purpose. These include the need to account for and robustly identify low frequency alleles, the non-uniformity of coverage and end bias [39], and the determination of adequate pooling and coverage to distinguish true SNPs from sequencing error [30], [43], [44]. Similarly, minimisation of the potential effects of PCR error during amplicon and library preparation [45] needs to be addressed. Accurate quantification of amplicons is also crucial to ensure all individuals comprising a pool are represented in equimolar amounts [43], [46], [47].

Site-specific analysis determined that SNPs identified from the Illumina data with an allele frequency greater than 0.5% were above the error threshold for this analysis. Subsequent Sequenom analysis confirmed the presence of SNP with an allele frequency as low as 0.7%. Similarly, Tsai *et al.* (2011) reliably identified heterozygous mutations in pools of 96 diploid individuals (allele frequency of 0.52%) and confirmed that utilizing a 0.5% minimum allele frequency greatly reduced the risk of false positives. Hence the lowest frequency SNPs identifiable from our 109 pools would be an individual with a homozygous SNP in a single genome or wild-type heterozygous loci on both (allele frequency of 0.46%), which is then expected to be slightly over-represented in the Illumina data [30]. As DNA from up to three M2 siblings was included in the pools, these SNPs may in fact occur at a frequency of up to 1.38%. Within our pool of 754 mutants, the homozygous SNP frequency for an individual (0.07%–0.20%) lies well below the imposed error threshold. Although we were able to identify some wild-type SNPs unique to this pool, there are likely to be more unidentified true SNPs (false negatives), which were disregarded due to their low frequency in the population.

As expected, the frequency and distribution of the 234 SNPs was gene dependent (Figure 5). This variation may be partially due to the different amplicon sizes, proportion of intron sequence screened and the sequence composition. With an abundance of wild-type variation (14.3 SNP/Mb), it is to be expected that the sub-sample represented in each pool would not capture all the variability in the base population. Since a relatively small number of control samples were screened, it is not surprising that we identified many SNPs unique to the mutant populations which are not the result of mutagenesis. The use of an LD_97_ may have created a genetic bottleneck amongst the mutant population. It is therefore to be expected that some non-EMS induced SNPs were unique to the control populations.

The wild-type polymorphism in the AR1 line (14.3 SNP/Mb) indicates that there is considerable diversity within the base material which has potential to be captured, though the greater proportion of these polymorphisms appear to be functionally neutral. If the estimated EMS induced mutation density generated in the *M. stipoides* population is accurate (2.4 mutations/Mb), it is lower than mutation densities previously reported for hexaploid (42 mutations/Mb) and tetraploid (25 mutations/Mb) wheat [41] and in mesopolyploid *Brassica* species (17 mutations/Mb) [48]. However our data is in accord with the tenet that polyploids are capable of withstanding high dose chemical mutation (LD_97_) without inducing significant levels of M2 lethality or sterility.

Many traits associated with the domestication syndrome are often the result of a loss of function of a recessive gene, such as seed shattering, which in the wild is advantageous and maintained by heterozygosity and natural selection [18]. Such genes are highly desirable targets when domesticating a new species. Both natural polymorphisms and induced mutations can cause such a loss of function of these genes, resulting in a ‘domesticated phenotype’. NGS facilitates the screening of large populations for polymorphisms which may induce loss of function. This approach can contribute to identification of candidate alleles for selection and pre-breeding programs.

The non-shattering phenotype was observed in the control lines, but was more prevalent in the mutant population. A non-shattering phenotype in rice can result from loss of function of either the *qSH1* gene, controlling the formation of the grain abscission layer, or the *sh4* gene, a putative transcription factor [19]–[21]. We identified a wild-type SNP in exon one of *qSH1*, putatively causing a premature stop codon. The *sh4* amplicon screened was 52% intronic DNA, and the majority of wild-type SNPs occurred in this non-coding region. However two SNPs (a C/A and a G/T) identified as causing putative premature stop codons were identified at the 5′ end of exon two. These SNPs may be responsible for the low numbers of non-shattering plants observed in the control lines. Subsequent screening using Sequenom MassARRAY analysis for the C/A SNP confirmed its presence in an individual recorded to have a non-shattering phenotype at harvest.

Loss of function of the *waxy* gene, encoding Granule Bound Starch Synthase I, causes high amylose (waxy) starch to form in grain endosperm in cultivated hexaploid wheat, where a gene dosage effect has been identified. This gene has now been shown to be the major determinant of endosperm starch composition in rice [49]. This was the only gene we examined in *M. stipoides* which had species-specific UTR based primers. As starch composition of the endosperm is important for seed germination, SNPs affecting this gene's function may be under strong selective pressure. Of the 49 SNPs identified in this gene the majority occurred in non-coding regions. Only two potentially non-synonymous SNPs were found, one early in the transit peptide and the other at the end of exon 13.

The *Isa* gene, first characterised in barley, encodes bi-functional amylase/subtilisin inhibitor which acts as part of a seed's defense mechanism against fungal and bacterial pathogens [50]. This locus is reported as a small single copy gene with no introns in rice, barley and wild barley (*Hordeum*) species. Sequence diversity within this gene has been positively correlated with increasing environmental variability [34], [51]. Only two of the five SNPs identified in the *Isa* gene of the wild populations were putatively functional, and neither would necessarily cause a loss of function [34]. Two non-synonymous SNPs identified in wild populations sampled close to the provenance of the AR1 base population were also identified in our AR1 control pool and/or the M2 754 pool. In both cases the minor allele from the wild population was the consensus sequence in the Illumina data.

In species where the breeding system is well understood, reverse genetic information provides the opportunity for a renaissance in mutation breeding, precisely because it can pin-point and isolate an independent series of component traits and their alleles. This information may then be used to guide breeding programs over a relatively short number of generations. Where pooled samples are analyzed using short read NGS technologies, it is critical that rare natural or mutant alleles are distinguishable from sequencing error [39], [43], [44], [46]. It is encouraging that we were able to identify unique SNPs in the pool of 754 M2 individuals. With the increasing throughput, read-length, specificity and sensitivity of NGS platforms, the associated reduction in error thresholds will contribute to more efficient and accurate screening of large mutant populations, attainable at greater pooling depths.

We have successfully accelerated the process of domestication in *M. stipoides* and demonstrated value in both forward and reverse genetic screening of the population. The reverse genetic screen has added valuable knowledge about the extent of the diversity within this base population, while screening only a very limited proportion of such a large genome (∼0.001%). Continued technological developments in DNA sequencing will allow greater efficacy of deep pooled screening, and genome-wide screening to include a comprehensive set of domestication genes. Although mutation breeding has been widely used over the past 80+ years to introduce novel alleles into many major crops, it has only had limited use in accelerated domestication. Here we have been able to select mutants with a set of component phenotypes representing multiple beneficial traits without the use of a cross breeding strategy. These phenotypes were identified during the forward screening of the mutant population and included beneficial combinations of plant size, improved architecture, compact panicle structure, increased seed size, non-shattering habit, rhizome production, increased tillering, shorter awn length, increased dry matter yield and greater seed production.

With the rapidly advancing field of molecular genomics and a growing understanding of the genetic events behind domestication, the utilization of molecular techniques in conjunction with mutation breeding should make it possible to accelerate the domestication of other wild plants as new environmentally sustainable crop species.

## Supporting Information

Figure S1 **EMS dosage effect on frequency of seed germination.** Note: a single fertile seedling was produced by the 175 mM EMS treatment, though there was complete lethality at both 160 mM and 200 mM EMS treatments.
(TIF)

Figure S2 **Sequence variability as measured by the method of Shenkin** ***et al.*** **(1991), for the** ***Microlaena stipoides waxy*** **gene.** The information-theoretical complexity measure *S* is plotted for each nucleotide position. **A**. Control pool; **B**. pool of 109 mutant individuals; **C**. pool of 754 mutant individuals.
(TIF)

File S1 **Table S1**: Primers and PCR conditions for gene homologues amplified for next generation SNP discovery. **Table S2**: Complete details of SNP loci identified in the four target genes in *Microlaena stipoides*, within the Illumina sequence data above the error threshold (an allele frequency >0.5%).
(DOCX)
