# Supplementary material for: High-Throughput Sequencing and Mutagenesis to Accelerate the Domestication of Microlaena stipoides as a New Food Crop
Source: PLoS One. 2013 Dec 18;8(12):e82641. doi: 10.1371/journal.pone.0082641 (PMC3867367; doi:10.1371/journal.pone.0082641)
Supplement: File S1 — Table S1: Primers and PCR conditions for gene homologues amplified for next generation SNP discovery. Table S2: Complete details of SNP loci identified in the four target genes in Microlaena stipoides, within the Illumina sequence data above the error threshold (an allele frequency >0.5%). (DOCX) [file pone.0082641.s003.docx]

**Table S1**: Primers and PCR conditions for gene homologues amplified for next generation SNP discovery

| **Gene Homologue** | **Genbank Accession for *M. stipoides* sequence** | **Primer Sequence 5'-3'** | **PCR Step 1**** Cycle # *Temp | **PCR Step 2**** Cycle # *Temp | **Polymerase** |
| --- | --- | --- | --- | --- | --- |
| *waxy* | EF600044 | F AAAATTCTGTTATCCCAACCC | 8 * 58-50ºC | 30 * 50ºC | Accuprime DG |
|  |  | R ATAATGGCTACGACAGACTCAC |  |  |  |
| *Isa* | HQ008272 | F TCCCCCTCATACTCCTCTCC | n/a | 35 * 66 ºC | Platinum Taq |
|  |  | R CACGAGTCCCTGCACGACAC |  |  |  |
| *qSH1* | HQ008270 | F CCCGGGTTCTATTCGTACGC | n/a | 35 * 58 ºC | Platinum Taq |
|  |  | R GCCCTCCAAATGAGCCCATC |  |  |  |
| *sh4*/SHA1 | HQ008271 | F CGCTCATCCTCATCACTGCCAA | 10 * 58-49ºC | 35 * 48 ºC | Roche Taq |
|  |  | R TCCCGCTTCTCCTCGCAC |  |  |  |

**Two step PCR was utilised: the first step a touchdown cycle, each cycle the annealing temperature decreasing one degree within the given range, the second step at a constant annealing temperature for the given number of cycles.

| **Table S2:** Complete details of SNP loci identified in the four target genes in *Microlaena stipoides*, within the Illumina sequence data above the error threshold (an allele frequency > 0.5%). | | | | | | | | | | | | | | |
| --- | --- | --- | --- | --- | --- | --- | --- | --- | --- | --- | --- | --- | --- | --- |
|  |  |  |  |  |  |  |  |  |  |  |  |  |  |  |
| **Pool** | **Gene** | **Reference position** | **Reference base** | **Allele variations** | **Coverage** | **Consensus from illumina reads** | **Frequency of concensus base** | **Count of consensus base** | **SNP** | **Frequency of SNP** | **Rare (R) or Common (C) SNP** | **Count of SNP** | ***In silico* Amino Acid Change** | **Synonymous (s), Non-synonymous (ns), or non-coding (nc) polymorphism** |
| m754 | *Isa* | 14 | C | C/T | 19,574 | C | 99.2800 | 19,433 | T | 0.6900 | R | 135 | Pro5Leu | ns |
| c109 | *Isa* | 14 | C | C/T | 22749 | C | 99.1428 | 22554 | T | 0.8088 | R | 184 | Pro5Leu | ns |
| m109 | *Isa* | 14 | C | C/T | 32089 | C | 99.0838 | 31795 | T | 0.8258 | R | 265 | Pro5Leu | ns |
| m754 | *Isa* | 61 | T | T/C | 779 | T | 99.2300 | 773 | C | 0.7700 | R | 6 | Phe21Leu | s |
| c109 | *Isa* | 231 | C | T/C | 4438 | T | 99.3466 | 4409 | C | 0.6534 | R | 29 |  | nc |
| m754 | *Isa* | 231 | C | T/C | 4,922 | T | 94.6770 | 4,660 | C | 5.3230 | C | 262 |  | nc |
| m754 | *Isa* | 302 | A | A/G | 962 | A | 99.4800 | 957 | G | 0.5200 | R | 5 | Glu101Gly | ns |
| m109 | *Isa* | 304 | A | A/G | 984 | A | 99.3902 | 978 | G | 0.5081 | R | 5 | Asn102Tyr | ns |
| m109 | *qSH1* | 80 | G | G/A | 62082 | G | 99.4121 | 61717 | A | 0.5525 | R | 343 | Ser26Asn | s |
| m109 | *qSH1* | 165 | G | G/T | 10264 | G | 99.2011 | 10182 | T | 0.7405 | R | 76 |  | nc |
| m754 | *qSH1* | 462 | C | C/G | 811 | C | 99.1370 | 804 | G | 0.8630 | R | 7 |  | nc |
| m754 | *qSH1* | 463 | T | T/C | 783 | T | 98.8510 | 774 | C | 1.0220 | R | 8 | Ser154Pro | ns |
| m754 | *qSH1* | 468 | T | T/C | 1,126 | T | 91.1190 | 1,026 | C | 8.7920 | C | 99 |  | nc |
| m754 | *qSH1* | 471 | A | A/G | 1,195 | A | 91.5480 | 1,094 | G | 8.3680 | C | 100 |  | nc |
| m754 | *qSH1* | 487 | T | T/G | 3,644 | T | 99.1490 | 3,613 | G | 0.6310 | R | 23 | Trp162Gly | ns |
| c109 | *qSH1* | 487 | T | T/G | 565 | T | 96.2832 | 544 | G | 3.0088 | C | 17 | Trp162Gly | ns |
| m754 | *qSH1* | 488 | G | G/A | 4,320 | G | 96.7820 | 4,181 | A | 3.0090 | C | 130 | Trp162Stp | premature stop |
| c109 | *qSH1* | 488 | G | G/A | 930 | G | 75.8065 | 705 | A | 23.9785 | C | 223 | Trp162Stp | premature stop |
| m754 | *qSH1* | 491 | A | A/T | 8,465 | A | 94.5540 | 8,004 | T | 0.6020 | R | 51 | Lys163Met | ns |
| c109 | *qSH1* | 491 | A | A/C | 4079 | A | 94.3123 | 3847 | C | 1.6180 | R | 66 | Lys163 |  |
| m754 | *qSH1* | 491 | A | A/G | 8,465 | A | 94.5540 | 8,004 | G | 3.7800 | C | 320 | Lys163Arg | ns |
| c109 | *qSH1* | 491 | A | A/G | 4079 | A | 94.3123 | 3847 | G | 3.8245 | C | 156 | Lys163Arg | ns |
| m109 | *qSH1* | 572 | T | T/C | 74426 | T | 99.0877 | 73747 | C | 0.6557 | R | 488 |  | nc |
| m109 | *qSH1* | 880 | T | G/T | 90503 | G | 67.2563 | 60869 | T | 32.6696 | C | 29567 |  | nc |
| m109 | *qSH1* | 1309 | T | C/T | 60052 | C | 66.1743 | 39739 | T | 33.7907 | C | 20292 | Tyr182Tyr | s |
| m109 | *qSH1* | 1472 | A | A/G | 39090 | A | 95.0550 | 37157 | G | 4.8913 | C | 1912 | Lys237Glu | ns |
| m109 | *qSH1* | 1473 | A | G/A | 38762 | G | 59.0630 | 22894 | A | 40.8880 | C | 15849 | Lys237Arg | s |
| m109 | *qSH1* | 1511 | G | C/G | 28343 | C | 66.9407 | 18973 | G | 32.9817 | C | 9348 | Ala250Pro | ns |
| m109 | *qSH1* | 2134 | G | A/G | 103322 | A | 67.4397 | 69680 | G | 32.5245 | C | 33605 |  | nc |
| m109 | *qSH1* | 2256 | A | T/A | 100446 | T | 64.8657 | 65155 | A | 34.9840 | C | 35140 |  | nc |
| m109 | *qSH1* | 2257 | T | A/T | 100425 | A | 64.8972 | 65173 | T | 34.9584 | C | 35107 |  | nc |
| m109 | *qSH1* | 2288 | N | A/C | 3484 | A | 95.6946 | 3334 | C | 3.3008 | C | 115 |  | nc |
| m109 | *qSH1* | 2418 | N | T/C | 4360 | T | 96.6972 | 4216 | C | 2.6835 | R | 117 |  | nc |
| m109 | *qSH1* | 2460 | G | G/A | 96646 | G | 70.9538 | 68574 | A | 28.9903 | C | 28018 |  | nc |
| m109 | *qSH1* | 3315 | A | A/G | 96298 | A | 99.4662 | 95784 | G | 0.5151 | R | 496 | Lys314Lys | s |
| m109 | *qSH1* | 3371 | T | T/C | 91227 | T | 71.7781 | 65481 | C | 28.1934 | C | 25720 |  | nc |
| m754 | *qSH1* | 3484 | G | G/T | 751 | G | 96.4050 | 724 | G | 3.5950 | C | 27 |  | nc |
| m109 | *qSH1* | 3556 | C | T/C | 69579 | T | 65.4982 | 45573 | C | 34.4601 | C | 23977 | Leu345Leu | s |
| m754 | *qSH1* | 3581 | C | C/A | 498 | C | 97.9920 | 488 | A | 2.0080 | R | 10 | Pro353His | ns |
| m754 | *qSH1* | 3583 | T | T/C | 526 | T | 99.0490 | 521 | C | 0.7600 | R | 4 | Ser354Pro | ns |
| m754 | *qSH1* | 3585 | T | T/A | 580 | T | 98.2760 | 570 | A | 1.7240 | R | 10 |  | nc |
| m754 | *qSH1* | 3587 | T | T/C | 591 | T | 99.3230 | 587 | C | 0.6770 | R | 4 | Val355Ala | s |
| m754 | *qSH1* | 3589 | G | G/C | 665 | G | 98.0450 | 652 | C | 1.9550 | R | 13 | Val356Leu | s |
| m754 | *qSH1* | 3594 | C | C/A | 756 | C | 97.8840 | 740 | A | 2.1160 | R | 16 | Asp357Glu | s |
| m754 | *qSH1* | 3602 | A | A/G | 942 | A | 98.7260 | 930 | G | 0.6370 | R | 6 | Gln360Arg | ns |
| m754 | *qSH1* | 3618 | T | T/A | 1,423 | T | 98.9460 | 1,408 | A | 0.9140 | R | 13 | His365Gln | ns |
| m754 | *qSH1* | 3645 | C | T/C | 2,136 | T | 99.4380 | 2,124 | C | 0.5150 | R | 11 | Ser374Ser | s |
| m109 | *qSH1* | 3645 | C | T/C | 66399 | T | 67.0356 | 44511 | C | 32.8815 | C | 21833 | Ser374Ser | s |
| m754 | *qSH1* | 3663 | T | T/C | 2,152 | T | 99.4420 | 2,140 | C | 0.5110 | R | 11 |  | nc |
| m754 | *qSH1* | 3666 | T | T/C | 2,070 | T | 99.3240 | 2,056 | C | 0.6280 | R | 13 |  | nc |
| m754 | *qSH1* | 3678 | T | T/C | 1,494 | T | 99.3310 | 1,484 | C | 0.6020 | R | 9 |  | nc |
| m754 | *qSH1* | 3707 | A | A/G | 1,679 | A | 99.1660 | 1,665 | G | 0.8340 | R | 14 | His395Arg | s |
| m754 | *qSH1* | 3708 | T | T/C | 1,641 | T | 99.3910 | 1,631 | C | 0.6090 | R | 10 |  | nc |
| m754 | *qSH1* | 3731 | T | T/A | 1,613 | T | 99.1940 | 1,600 | A | 0.5580 | R | 9 | Phe403Tyr | s |
| m754 | *qSH1* | 3756 | C | C/A | 892 | C | 99.3270 | 886 | A | 0.6730 | R | 6 |  | nc |
| m754 | *qSH1* | 3768 | A | A/G | 1,050 | A | 97.6190 | 1,025 | G | 2.1900 | R | 23 |  | nc |
| m754 | *qSH1* | 3784 | G | G/T | 1,976 | G | 98.7350 | 1,951 | T | 1.2150 | R | 24 | Ala421Ser | ns |
| m754 | *qSH1* | 3786 | A | A/T | 2,117 | A | 98.8190 | 2,092 | T | 1.1810 | R | 25 |  | nc |
| m754 | *qSH1* | 3792 | C | C/T | 2,784 | C | 99.2100 | 2,762 | T | 0.7900 | R | 22 |  | nc |
| m754 | *qSH1* | 3795 | A | A/G | 3,065 | A | 98.5640 | 3,021 | G | 1.4360 | R | 44 |  | nc |
| m754 | *qSH1* | 3796 | T | T/A | 3,182 | T | 99.3400 | 3,161 | A | 0.5970 | R | 19 | Leu425Met | s |
| m754 | *qSH1* | 3817 | C | C/G | 4,121 | C | 98.0100 | 4,039 | G | 1.8440 | R | 76 | Pro432Ala | ns |
| m754 | *qSH1* | 3818 | C | C/T | 4,140 | C | 98.2610 | 4,068 | T | 1.6430 | R | 68 | Pro432Leu | ns |
| m754 | *qSH1* | 3822 | T | T/A | 3,902 | T | 98.0520 | 3,826 | A | 1.8200 | R | 71 |  | nc |
| m754 | *qSH1* | 3838 | G | G/T | 3,107 | G | 98.3260 | 3,055 | T | 1.6410 | R | 51 | Ala439Ser | ns |
| m754 | *qSH1* | 3843 | A | A/G | 2,889 | A | 98.2690 | 2,839 | G | 1.7310 | R | 50 |  | nc |
| m754 | *qSH1* | 3847 | T | T/C | 2,636 | T | 98.2550 | 2,590 | C | 1.6690 | R | 44 | Tyr442His | ns |
| m754 | *qSH1* | 3850 | G | G/A | 2,409 | G | 97.8830 | 2,358 | A | 2.0760 | R | 50 | Asp443Asn | ns |
| m754 | *qSH1* | 3877 | C | C/G | 1,301 | C | 98.0020 | 1,275 | G | 1.9220 | R | 25 | Gln452Glu | nc |
| m754 | *qSH1* | 3881 | C | C/T | 1,113 | C | 97.9340 | 1,090 | T | 1.9770 | R | 22 | Ala453Val | s |
| m754 | *qSH1* | 3883 | A | A/G | 1,031 | A | 97.6720 | 1,007 | G | 2.2310 | R | 23 | Ser454Gly | nc |
| m754 | *qSH1* | 3897 | C | C/T | 809 | C | 98.0220 | 793 | T | 1.8540 | R | 15 | Asn458Asn | s |
| m754 | *qSH1* | 3900 | C | C/T | 787 | C | 85.0060 | 669 | T | 14.8670 | C | 117 |  | nc |
| m109 | *qSH1* | 3900 | C | C/T | 16394 | C | 72.8803 | 11948 | T | 27.0770 | C | 4439 |  | nc |
| m754 | *qSH1* | 3903 | T | T/C | 770 | T | 97.9220 | 754 | C | 2.0780 | R | 16 |  | nc |
| m754 | *qSH1* | 3905 | T | T/C | 754 | T | 99.4690 | 750 | C | 0.5310 | R | 4 | Val461Ala | s |
| m754 | *qSH1* | 3911 | T | T/C | 744 | T | 99.0590 | 737 | C | 0.6720 | R | 5 | Leu463Pro | ns |
| m754 | *qSH1* | 3996 | T | T/C | 2,772 | T | 96.9340 | 2,687 | C | 2.9580 | C | 82 | Phe491Phe | s |
| c109 | *qSH1* | 4016 | A | A/C | 843 | A | 98.5765 | 831 | C | 1.0676 | R | 9 |  | nc |
| c109 | *qSH1* | 4021 | T | T/C | 1042 | T | 95.6814 | 997 | C | 4.3186 | C | 45 |  | nc |
| c109 | *qSH1* | 4022 | A | A/T | 1826 | A | 97.4808 | 1780 | T | 2.4096 | R | 44 |  | nc |
| m754 | *qSH1* | 4025 | T | T/A | 10,373 | T | 97.3970 | 10,103 | A | 1.7450 | R | 181 | Val501Glu | ns |
| c109 | *qSH1* | 4025 | T | T/A | 8764 | T | 95.4815 | 8368 | A | 3.6855 | C | 323 | Val501Glu | ns |
| m109 | *sh4* | 16 | G | G/C | 789 | G | 97.8454 | 772 | C | 1.9011 | R | 15 | Ala6Pro | ns |
| c109 | *sh4* | 18 | G | G/A | 899 | G | 99.3326 | 893 | A | 0.5562 | R | 5 |  | nc |
| m109 | *sh4* | 18 | G | G/A | 940 | G | 98.4043 | 925 | A | 1.5957 | R | 15 |  | nc |
| c109 | *sh4* | 27 | A | A/G | 1007 | A | 97.9146 | 986 | G | 2.0854 | R | 21 |  | nc |
| m109 | *sh4* | 27 | A | A/G | 1257 | A | 96.3405 | 1211 | G | 3.6595 | C | 46 |  | nc |
| m754 | *sh4* | 61 | T | T/C | 1,662 | T | 99.1580 | 1,648 | C | 0.8420 | R | 14 | Trp21Arg | ns |
| c109 | *sh4* | 61 | T | T/C | 3874 | T | 98.8126 | 3828 | C | 1.1100 | R | 43 | Trp21Arg | ns |
| m754 | *sh4* | 69 | G | G/A | 1,691 | G | 96.0970 | 1,625 | A | 3.9030 | C | 66 | Glu23Glu | s |
| c109 | *sh4* | 69 | G | G/A | 3825 | G | 94.0131 | 3596 | A | 5.9085 | C | 226 | Glu23Glu | s |
| m109 | *sh4* | 69 | G | G/A | 5812 | G | 92.0681 | 5351 | A | 7.9147 | C | 460 | Glu23Glu | s |
| m754 | *sh4* | 98 | G | G/A | 1,922 | G | 97.5550 | 1,875 | A | 2.3410 | R | 45 | Arg33His | s |
| c109 | *sh4* | 115 | A | A/G | 7115 | A | 97.6950 | 6951 | G | 2.2628 | R | 161 | Asn39Asp | ns |
| m754 | *sh4* | 115 | A | A/G | 4,071 | A | 96.4140 | 3,925 | G | 3.5860 | C | 146 | Asn39Asp | ns |
| c109 | *sh4* | 117 | C | C/G | 7402 | C | 97.8925 | 7246 | G | 2.0265 | R | 150 | Asn39Lys | ns |
| m754 | *sh4* | 117 | C | C/G | 4,267 | C | 96.5550 | 4,120 | G | 3.3280 | C | 142 | Asn39Lys | ns |
| c109 | *sh4* | 120 | C | C/G | 7603 | C | 98.0008 | 7451 | G | 1.9598 | R | 149 | Asp40Glu | s |
| m754 | *sh4* | 120 | C | C/G | 4,457 | C | 96.7690 | 4,313 | G | 3.2080 | C | 143 | Asp40Glu | s |
| c109 | *sh4* | 124 | T | T/C | 7440 | T | 97.7151 | 7270 | C | 2.2446 | R | 167 | Trp42Arg | ns |
| m754 | *sh4* | 124 | T | T/C | 4,428 | T | 96.5900 | 4,277 | C | 3.3200 | C | 147 | Trp42Arg | ns |
| m109 | *sh4* | 138 | C | C/G | 31526 | C | 97.5449 | 30752 | G | 2.3853 | R | 752 |  | nc |
| m754 | *sh4* | 138 | C | C/G | 7,125 | C | 96.3090 | 6,862 | G | 3.6070 | C | 257 |  | nc |
| c109 | *sh4* | 138 | C | C/G | 9984 | C | 93.0288 | 9288 | G | 6.8910 | C | 688 |  | nc |
| m109 | *sh4* | 180 | A | A/C | 22320 | A | 88.1183 | 19668 | C | 11.7876 | C | 2631 |  | nc |
| m754 | *sh4* | 180 | A | A/C | 4,685 | A | 88.0900 | 4,127 | C | 11.8250 | C | 554 |  | nc |
| c109 | *sh4* | 180 | A | A/C | 8094 | A | 71.5962 | 5795 | C | 28.3173 | C | 2292 |  | nc |
| m754 | *sh4* | 189 | C | C/A | 3,171 | C | 99.4640 | 3,154 | A | 0.5360 | R | 17 |  | nc |
| m109 | *sh4* | 189 | C | C/A | 16570 | C | 98.5697 | 16333 | A | 1.3941 | R | 231 |  | nc |
| c109 | *sh4* | 189 | C | C/A | 9987 | C | 97.5568 | 9743 | A | 2.3330 | R | 233 |  | nc |
| c109 | *sh4* | 226 | C | C/T | 10414 | C | 99.1550 | 10326 | T | 0.8258 | R | 86 | Pro76Ser | ns |
| m109 | *sh4* | 226 | C | C/T | 5887 | C | 95.6514 | 5631 | T | 4.3316 | C | 255 | Pro76Ser | ns |
| m754 | *sh4* | 237 | T | T/C | 1,376 | T | 99.2010 | 1,365 | C | 0.7990 | R | 11 |  | nc |
| c109 | *sh4* | 237 | T | T/C | 6388 | T | 98.4346 | 6288 | C | 1.5185 | R | 97 |  | nc |
| m109 | *sh4* | 237 | T | T/C | 5985 | T | 96.3241 | 5765 | C | 3.6424 | C | 218 |  | nc |
| m754 | *sh4* | 238 | G | G/A | 1,413 | G | 99.3630 | 1,404 | A | 0.6370 | R | 9 | Asp80Asn | ns |
| c109 | *sh4* | 238 | G | G/A | 6061 | G | 98.5481 | 5973 | A | 1.4519 | R | 88 | Asp80Asn | ns |
| m109 | *sh4* | 238 | G | G/A | 6281 | G | 96.5451 | 6064 | A | 3.4071 | C | 214 | Asp80Asn | ns |
| m754 | *sh4* | 239 | A | A/G | 1,375 | A | 99.1270 | 1,363 | G | 0.8000 | R | 11 | Asp80Gly | ns |
| c109 | *sh4* | 239 | A | A/G | 5617 | A | 98.3799 | 5526 | G | 1.6201 | R | 91 | Asp80Gly | ns |
| m109 | *sh4* | 239 | A | A/G | 6252 | A | 96.5131 | 6034 | G | 3.4389 | C | 215 | Asp80Gly | ns |
| m754 | *sh4* | 259 | T | T/C | 2,207 | T | 99.2750 | 2,191 | C | 0.7250 | R | 16 |  | nc |
| c109 | *sh4* | 259 | T | T/C | 5091 | T | 98.3500 | 5007 | C | 1.5714 | R | 80 | Leu87Leu | s |
| m109 | *sh4* | 259 | T | T/C | 8693 | T | 97.8949 | 8510 | C | 2.0706 | R | 180 | Leu87Leu | s |
| m754 | *sh4* | 262 | G | G/C | 2,265 | G | 99.4700 | 2,253 | C | 0.5300 | R | 12 | Glu88Gln | s |
| c109 | *sh4* | 262 | G | G/C | 5147 | G | 98.3486 | 5062 | C | 1.5737 | R | 81 | Glu88Gln | s |
| m109 | *sh4* | 262 | G | G/C | 8754 | G | 98.0238 | 8581 | C | 1.8963 | R | 166 | Glu88Gln | s |
| c109 | *sh4* | 264 | G | G/A | 5445 | G | 98.5675 | 5367 | A | 1.4325 | R | 78 |  | nc |
| m754 | *sh4* | 264 | G | G/A | 2,474 | G | 99.3530 | 2,458 | A | 0.6060 | R | 15 | Glu88Glu | s |
| m109 | *sh4* | 264 | G | G/A | 9483 | G | 98.3655 | 9328 | A | 1.5923 | R | 151 | Glu88Glu | s |
| m754 | *sh4* | 273 | A | A/G | 2,724 | A | 99.0460 | 2,698 | G | 0.9180 | R | 25 | Glu91Glu | s |
| m109 | *sh4* | 273 | A | A/G | 10501 | A | 98.3716 | 10330 | G | 1.5998 | R | 168 | Glu91Glu | s |
| c109 | *sh4* | 273 | A | A/G | 6052 | A | 98.3311 | 5951 | G | 1.6358 | R | 99 | Glu91Glu | s |
| m754 | *sh4* | 280 | A | A/G | 2,785 | A | 99.1380 | 2,761 | G | 0.8620 | R | 24 | Asn94Asp | ns |
| c109 | *sh4* | 280 | A | A/G | 6316 | A | 98.7492 | 6237 | G | 1.2508 | R | 79 | Asn94Asp | ns |
| m109 | *sh4* | 280 | A | A/G | 10699 | A | 98.7102 | 10561 | G | 1.2805 | R | 137 | Asn94Asp | ns |
| m754 | *sh4* | 294 | A | A/C | 2,669 | A | 99.0630 | 2,644 | C | 0.8990 | R | 24 |  | nc |
| c109 | *sh4* | 294 | A | A/C | 7020 | A | 98.8746 | 6941 | C | 0.9402 | R | 66 |  | nc |
| m109 | *sh4* | 294 | A | A/C | 10842 | A | 98.7179 | 10703 | C | 1.1621 | R | 126 |  | nc |
| m754 | *sh4* | 336 | G | G/C | 1,509 | G | 99.3370 | 1,499 | C | 0.5960 | R | 9 |  | nc |
| m109 | *sh4* | 336 | G | G/C | 8357 | G | 98.4803 | 8230 | C | 1.4838 | R | 124 |  | nc |
| c109 | *sh4* | 336 | G | G/C | 6148 | G | 98.3409 | 6046 | C | 1.6103 | R | 99 |  | nc |
| m109 | *sh4* | 345 | T | T/C | 7301 | T | 98.3427 | 7180 | C | 1.5888 | R | 116 |  | nc |
| c109 | *sh4* | 345 | T | T/C | 5404 | T | 98.0940 | 5301 | C | 1.8505 | R | 100 |  | nc |
| m754 | *sh4* | 368 | T | T/C | 917 | T | 98.0370 | 899 | C | 1.9630 | R | 18 | Val123Ala | s |
| c109 | *sh4* | 368 | T | T/C | 4118 | T | 96.4546 | 3972 | C | 3.5211 | C | 145 | Val123Ala | s |
| m109 | *sh4* | 368 | T | T/C | 6145 | T | 96.1269 | 5907 | C | 3.8731 | C | 238 | Val123Ala | s |
| m754 | *sh4* | 384 | G | G/C | 1,253 | G | 98.9620 | 1,240 | C | 0.8780 | R | 11 |  | nc |
| c109 | *sh4* | 384 | G | G/C | 5622 | G | 97.9545 | 5507 | C | 1.9032 | R | 107 |  | nc |
| m109 | *sh4* | 384 | G | G/C | 8366 | G | 97.8126 | 8183 | C | 2.0798 | R | 174 |  | nc |
| c109 | *sh4* | 390 | A | A/T | 5443 | A | 98.2914 | 5350 | T | 1.6351 | R | 89 |  | nc |
| m109 | *sh4* | 390 | A | A/T | 7911 | A | 97.7752 | 7735 | T | 2.1615 | R | 171 |  | nc |
| c109 | *sh4* | 405 | G | G/A | 7798 | G | 99.0126 | 7721 | A | 0.9746 | R | 76 |  | nc |
| m109 | *sh4* | 405 | G | G/A | 12356 | G | 98.3814 | 12156 | A | 1.6025 | R | 198 |  | nc |
| m109 | *sh4* | 411 | T | T/G | 14264 | T | 99.2639 | 14159 | G | 0.6450 | R | 92 |  | nc |
| m109 | *sh4* | 414 | T | T/G | 15578 | T | 98.9408 | 15413 | G | 0.9950 | R | 155 |  | nc |
| c109 | *sh4* | 414 | T | T/G | 11434 | T | 98.9155 | 11310 | G | 1.0495 | R | 120 |  | nc |
| c109 | *sh4* | 429 | G | G/A | 14959 | G | 98.1215 | 14678 | A | 1.8584 | R | 278 |  | nc |
| m754 | *sh4* | 435 | T | T/G | 4,077 | T | 99.3130 | 4,049 | G | 0.5640 | R | 23 |  | nc |
| c109 | *sh4* | 435 | T | T/G | 15456 | T | 97.6967 | 15100 | G | 2.1610 | R | 334 |  | nc |
| m109 | *sh4* | 435 | T | T/G | 19188 | T | 97.5193 | 18712 | G | 2.3035 | R | 442 |  | nc |
| m754 | *sh4* | 438 | A | A/G | 3,885 | A | 99.3820 | 3,861 | G | 0.5660 | R | 22 |  | nc |
| c109 | *sh4* | 438 | A | A/G | 14595 | A | 97.7869 | 14272 | G | 2.1377 | R | 312 |  | nc |
| m109 | *sh4* | 438 | A | A/G | 17460 | A | 97.6460 | 17049 | G | 2.3368 | R | 408 |  | nc |
| c109 | *sh4* | 441 | A | A/G | 15942 | A | 97.8547 | 15600 | G | 2.1202 | R | 338 | Lys147Lys | s |
| m109 | *sh4* | 441 | A | A/G | 19052 | A | 97.6328 | 18601 | G | 2.3620 | R | 450 | Lys147Lys | s |
| m754 | *sh4* | 446 | A | A/T | 4,580 | A | 99.3890 | 4,552 | T | 0.5680 | R | 26 | His149Leu | ns |
| c109 | *sh4* | 446 | A | A/T | 16599 | A | 97.3010 | 16151 | T | 2.5604 | R | 425 | His149Leu | ns |
| m109 | *sh4* | 446 | A | A/T | 19363 | A | 97.2422 | 18829 | T | 2.6132 | R | 506 | His149Leu | ns |
| m754 | *sh4* | 455 | A | A/G | 4,488 | A | 82.9320 | 3,722 | G | 17.0010 | C | 763 | Gln152Arg | ns |
| m109 | *sh4* | 455 | A | A/G | 18469 | A | 80.5620 | 14879 | G | 19.3784 | C | 3579 | Gln152Arg | ns |
| c109 | *sh4* | 455 | A | A/G | 16397 | A | 65.2314 | 10696 | G | 34.7259 | C | 5694 | Gln152Arg | ns |
| m109 | *sh4* | 468 | A | A/G | 18500 | A | 98.8757 | 18292 | G | 1.1081 | R | 205 |  | nc |
| c109 | *sh4* | 468 | A | A/G | 17302 | A | 98.7631 | 17088 | G | 1.1617 | R | 201 |  | nc |
| m109 | *sh4* | 470 | T | T/C | 17588 | T | 98.6695 | 17354 | C | 1.3020 | R | 229 | Leu157Pro | ns |
| c109 | *sh4* | 470 | T | T/C | 16154 | T | 98.5205 | 15915 | C | 1.4609 | R | 236 | Leu157Pro | ns |
| m109 | *sh4* | 476 | A | A/C | 17323 | A | 98.8801 | 17129 | C | 1.0160 | R | 176 | Gln159Pro | ns |
| c109 | *sh4* | 476 | A | A/C | 15874 | A | 98.7086 | 15669 | C | 1.1276 | R | 179 | Gln159Pro | ns |
| m109 | *sh4* | 486 | T | T/C | 14130 | T | 85.8882 | 12136 | C | 14.0623 | C | 1987 |  | nc |
| m754 | *sh4* | 486 | T | T/C | 3,360 | T | 85.0890 | 2,859 | C | 14.9110 | C | 501 |  | nc |
| c109 | *sh4* | 486 | T | T/C | 12790 | T | 66.8491 | 8550 | C | 33.1431 | C | 4239 |  | nc |
| m109 | *sh4* | 489 | T | T/C | 13922 | T | 99.3607 | 13833 | C | 0.6105 | R | 85 |  | nc |
| c109 | *sh4* | 489 | T | T/C | 12805 | T | 99.1722 | 12699 | C | 0.8122 | R | 104 |  | nc |
| m109 | *sh4* | 492 | G | G/A | 11731 | G | 99.2754 | 11646 | A | 0.6564 | R | 77 |  | nc |
| c109 | *sh4* | 492 | G | G/A | 11532 | G | 99.1068 | 11429 | A | 0.8672 | R | 100 |  | nc |
| m109 | *sh4* | 511 | A | A/G | 15613 | A | 99.3403 | 15510 | G | 0.6405 | R | 100 | Thr171Ala | ns |
| c109 | *sh4* | 517 | G | G/A | 14783 | G | 98.3427 | 14538 | A | 1.6370 | R | 242 | Val173Ile | ns |
| m109 | *sh4* | 517 | G | G/A | 21982 | G | 96.5017 | 21213 | A | 3.4801 | C | 765 | Val173Ile | ns |
| c109 | *sh4* | 522 | C | C/T | 15936 | C | 98.4438 | 15688 | T | 1.5248 | R | 243 |  | nc |
| m109 | *sh4* | 522 | C | C/T | 24275 | C | 96.2966 | 23376 | T | 3.6498 | C | 886 |  | nc |
| c109 | *sh4* | 523 | G | G/C | 15782 | G | 98.4096 | 15531 | C | 1.5017 | R | 237 | Gly175Arg | ns |
| m109 | *sh4* | 523 | G | G/C | 24164 | G | 96.2258 | 23252 | C | 3.6128 | C | 873 | Gly175Arg | ns |
| m754 | *sh4* | 529 | G | G/A | 5,456 | G | 98.8640 | 5,394 | A | 1.1360 | R | 62 | Val177Ile | s |
| c109 | *sh4* | 529 | G | G/A | 17741 | G | 96.0036 | 17032 | A | 3.9738 | C | 705 | Val177Ile | s |
| m109 | *sh4* | 529 | G | G/A | 27398 | G | 92.2476 | 25274 | A | 7.7159 | C | 2114 | Val177Ile | s |
| m754 | *sh4* | 536 | C | C/A | 6,342 | C | 97.9970 | 6,215 | A | 1.9870 | R | 126 | Ala179Asp | ns |
| c109 | *sh4* | 536 | C | C/A | 19336 | C | 94.9990 | 18369 | A | 4.9648 | C | 960 | Ala179Asp | ns |
| m109 | *sh4* | 536 | C | C/A | 30587 | C | 89.9042 | 27499 | A | 10.0533 | C | 3075 | Ala179Asp | ns |
| m754 | *sh4* | 560 | T | T/C | 8,705 | T | 98.1160 | 8,541 | C | 1.8270 | R | 159 |  | nc |
| c109 | *sh4* | 560 | T | T/C | 23871 | T | 95.2746 | 22743 | C | 4.6668 | C | 1114 |  | nc |
| m109 | *sh4* | 560 | T | T/C | 37350 | T | 91.8929 | 34322 | C | 8.0535 | C | 3008 |  | nc |
| m754 | *sh4* | 562 | C | C/T | 8,814 | C | 98.2750 | 8,662 | T | 1.7130 | R | 151 |  | nc |
| c109 | *sh4* | 562 | C | C/T | 24153 | C | 95.5078 | 23068 | T | 4.4715 | C | 1080 |  | nc |
| m109 | *sh4* | 562 | C | C/T | 37540 | C | 92.1604 | 34597 | T | 7.7997 | C | 2928 |  | nc |
| m109 | *sh4* | 594 | A | A/G | 36688 | A | 99.4140 | 36473 | G | 0.5642 | R | 207 |  | nc |
| c109 | *sh4* | 594 | A | A/G | 25811 | A | 99.3414 | 25641 | G | 0.6431 | R | 166 |  | nc |
| m754 | *sh4* | 610 | A | A/G | 9,614 | A | 98.9290 | 9,511 | G | 1.0610 | R | 102 |  | nc |
| m109 | *sh4* | 610 | A | A/G | 39590 | A | 98.1889 | 38873 | G | 1.7757 | R | 703 |  | nc |
| c109 | *sh4* | 610 | A | A/G | 27741 | A | 97.7002 | 27103 | G | 2.2854 | R | 634 |  | nc |
| m109 | *sh4* | 626 | A | A/C | 40678 | A | 99.2256 | 40363 | C | 0.7105 | R | 289 |  | nc |
| c109 | *sh4* | 626 | A | A/C | 30110 | A | 98.7247 | 29726 | C | 1.0794 | R | 325 |  | nc |
| c109 | *sh4* | 632 | G | G/A | 31260 | G | 99.3154 | 31046 | A | 0.6718 | R | 210 |  | nc |
| c109 | *sh4* | 634 | C | C/T | 32125 | C | 99.3494 | 31916 | T | 0.6288 | R | 202 |  | nc |
| m109 | *sh4* | 653 | T | T/G/ | 42944 | T | 98.7845 | 42422 | G | 1.0712 | R | 460 |  | nc |
| m109 | *sh4* | 654 | T | T/A | 43950 | T | 98.8077 | 43426 | A | 1.0512 | R | 462 |  | nc |
| m109 | *sh4* | 655 | G | G/A | 44346 | G | 98.8680 | 43844 | A | 1.0756 | R | 477 |  | nc |
| m109 | *sh4* | 656 | C | C/T | 43321 | C | 98.8320 | 42815 | T | 1.1265 | R | 488 |  | nc |
| m109 | *sh4* | 672 | C | C/G | 46383 | C | 98.3270 | 45607 | G | 1.6062 | R | 745 |  | nc |
| m754 | *sh4* | 717 | G | G/A | 12,038 | G | 99.4270 | 11,969 | A | 0.5320 | R | 64 |  | nc |
| m754 | *sh4* | 741 | G | G/T | 12,209 | G | 99.3530 | 12,130 | T | 0.6390 | R | 78 |  | nc |
| m109 | *sh4* | 741 | G | G/T | 49088 | G | 95.0579 | 46662 | T | 4.8933 | C | 2402 |  | nc |
| c109 | *sh4* | 741 | G | G/T | 42376 | G | 92.9772 | 39400 | T | 6.9591 | C | 2949 |  | nc |
| m109 | *sh4* | 798 | A | A/C | 43600 | A | 83.1399 | 36249 | C | 16.7500 | C | 7303 |  | nc |
| m754 | *sh4* | 798 | A | A/C | 11,639 | A | 81.3130 | 9,464 | C | 18.5500 | C | 2159 |  | nc |
| c109 | *sh4* | 798 | A | A/C | 37982 | A | 65.0861 | 24721 | C | 34.7823 | C | 13211 |  | nc |
| m109 | *sh4* | 814 | T | T/C | 49581 | T | 99.4655 | 49316 | C | 0.5163 | R | 256 |  | nc |
| c109 | *sh4* | 814 | T | T/C | 43651 | T | 99.3173 | 43353 | C | 0.6437 | R | 281 |  | nc |
| m754 | *sh4* | 827 | T | T/G | 12,446 | T | 98.9800 | 12,319 | G | 0.5540 | R | 69 |  | nc |
| m109 | *sh4* | 827 | T | T/A | 50777 | T | 99.1433 | 50342 | A | 0.7208 | R | 366 |  | nc |
| c109 | *sh4* | 827 | T | T/A | 44728 | T | 98.0348 | 43849 | A | 1.8199 | R | 814 |  | nc |
| c109 | *sh4* | 852 | A | A/G | 46458 | A | 98.2543 | 45647 | G | 1.7241 | R | 801 |  | nc |
| c109 | *sh4* | 855 | A | A/G | 46299 | A | 98.0453 | 45394 | G | 1.9309 | R | 894 |  | nc |
| c109 | *sh4* | 856 | G | G/A | 46834 | G | 98.2342 | 46007 | A | 1.7509 | R | 820 |  | nc |
| m754 | *sh4* | 883 | T | T/C | 14,367 | T | 99.3390 | 14,272 | C | 0.6330 | R | 91 |  | nc |
| m109 | *sh4* | 888 | G | G/A | 55022 | G | 98.9695 | 54455 | A | 1.0141 | R | 558 |  | nc |
| c109 | *sh4* | 888 | G | G/A | 46956 | G | 98.6093 | 46303 | A | 1.3715 | R | 644 |  | nc |
| c109 | *sh4* | 897 | T | T/A | 48045 | T | 98.9572 | 47544 | A | 0.9512 | R | 457 |  | nc |
| m109 | *sh4* | 897 | T | T/A | 55278 | T | 98.8911 | 54665 | A | 1.0492 | R | 580 |  | nc |
| c109 | *sh4* | 902 | T | T/C | 47123 | T | 98.9814 | 46643 | C | 1.0080 | R | 475 |  | nc |
| m109 | *sh4* | 902 | T | T/C | 53965 | T | 98.6936 | 53260 | C | 1.2990 | R | 701 |  | nc |
| c109 | *sh4* | 907 | G | G/A | 47202 | G | 99.1102 | 46782 | A | 0.8750 | R | 413 |  | nc |
| m109 | *sh4* | 907 | G | G/A | 53933 | G | 98.7726 | 53271 | A | 1.1996 | R | 647 |  | nc |
| c109 | *sh4* | 909 | T | T/A | 47375 | T | 98.9910 | 46897 | A | 0.9013 | R | 427 |  | nc |
| m109 | *sh4* | 909 | T | T/A | 54099 | T | 98.6802 | 53385 | A | 1.2181 | R | 659 |  | nc |
| c109 | *sh4* | 920 | G | G/C | 46467 | G | 99.2123 | 46101 | C | 0.7145 | R | 332 |  | nc |
| m109 | *sh4* | 920 | G | G/C | 54341 | G | 98.9308 | 53760 | C | 0.9919 | R | 539 |  | nc |
| c109 | *sh4* | 924 | A | A/G | 46022 | A | 99.2286 | 45667 | G | 0.7518 | R | 346 |  | nc |
| m109 | *sh4* | 924 | A | A/G | 54505 | A | 98.9781 | 53948 | G | 1.0128 | R | 552 |  | nc |
| m109 | *sh4* | 946 | G | G/C | 51689 | G | 83.7025 | 43265 | C | 16.2123 | C | 8380 |  | nc |
| m754 | *sh4* | 946 | G | G/C | 13,295 | G | 81.9560 | 10,896 | C | 17.9990 | C | 2393 |  | nc |
| c109 | *sh4* | 946 | G | G/C | 41763 | G | 63.9561 | 26710 | C | 35.9648 | C | 15020 |  | nc |
| c109 | *sh4* | 954 | C | C/T | 40756 | C | 99.3866 | 40506 | T | 0.5962 | R | 243 |  | nc |
| m109 | *sh4* | 954 | C | C/T | 51555 | C | 99.2784 | 51183 | T | 0.6905 | R | 356 |  | nc |
| m754 | *sh4* | 965 | G | G/T | 12,900 | G | 99.0850 | 12,782 | T | 0.8760 | R | 113 |  | nc |
| c109 | *sh4* | 965 | G | G/T | 39622 | G | 97.9178 | 38797 | T | 2.0166 | R | 799 |  | nc |
| m109 | *sh4* | 965 | G | G/T | 51040 | G | 96.8123 | 49413 | T | 3.1289 | C | 1597 |  | nc |
| m754 | *sh4* | 975 | T | T/C | 13,150 | T | 98.2050 | 12,914 | C | 1.7490 | R | 230 |  | nc |
| c109 | *sh4* | 975 | T | T/C | 40055 | T | 97.0615 | 38878 | C | 2.8860 | C | 1156 |  | nc |
| m109 | *sh4* | 975 | T | T/C | 52667 | T | 95.9557 | 50537 | C | 4.0101 | C | 2112 |  | nc |
| m754 | *sh4* | 993 | C | C/T | 13,289 | C | 97.6820 | 12,981 | T | 2.2880 | R | 304 |  | nc |
| c109 | *sh4* | 993 | C | C/T | 39320 | C | 96.2792 | 37857 | T | 3.6979 | C | 1454 |  | nc |
| m109 | *sh4* | 993 | C | C/T | 53342 | C | 94.9739 | 50661 | T | 4.9961 | C | 2665 |  | nc |
| m754 | *sh4* | 997 | A | A/C | 13,245 | A | 97.5390 | 12,919 | C | 2.2800 | R | 302 |  | nc |
| c109 | *sh4* | 997 | A | A/C | 39382 | A | 95.8788 | 37759 | C | 3.9383 | C | 1551 |  | nc |
| m109 | *sh4* | 997 | A | A/C | 53820 | A | 94.5373 | 50880 | C | 5.3214 | C | 2864 |  | nc |
| m754 | *sh4* | 1004 | G | G/T | 13,565 | G | 97.6040 | 13,240 | T | 2.3220 | R | 315 |  | nc |
| c109 | *sh4* | 1004 | G | G/T | 41705 | G | 94.6721 | 39483 | T | 5.2680 | C | 2197 |  | nc |
| m109 | *sh4* | 1004 | G | G/T | 56063 | G | 93.6375 | 52496 | T | 6.3001 | C | 3532 |  | nc |
| m754 | *sh4* | 1027 | C | C/A | 11,726 | C | 97.7060 | 11,457 | A | 2.2510 | R | 264 |  | nc |
| m109 | *sh4* | 1027 | C | C/A | 49173 | C | 92.7643 | 45615 | A | 7.2092 | C | 3545 |  | nc |
| c109 | *sh4* | 1027 | C | C/A | 40279 | C | 92.4253 | 37228 | A | 7.5424 | C | 3038 |  | nc |
| m754 | *sh4* | 1040 | T | T/A | 11,109 | T | 98.2990 | 10,920 | A | 1.6020 | R | 178 |  | nc |
| m109 | *sh4* | 1040 | T | T/A | 45774 | T | 93.0332 | 42585 | A | 6.8598 | C | 3140 |  | nc |
| c109 | *sh4* | 1040 | T | T/A | 40544 | T | 92.9114 | 37670 | A | 6.9727 | C | 2827 |  | nc |
| m754 | *sh4* | 1041 | T | T/C | 11,359 | T | 98.3270 | 11,169 | C | 1.6640 | R | 189 |  | nc |
| c109 | *sh4* | 1041 | T | T/C | 42134 | T | 93.1552 | 39250 | C | 6.8235 | C | 2875 |  | nc |
| m109 | *sh4* | 1041 | T | T/C | 47029 | T | 93.1447 | 43805 | C | 6.8405 | C | 3217 |  | nc |
| m754 | *sh4* | 1055 | A | A/T | 11,488 | A | 98.4680 | 11,312 | T | 1.2530 | R | 144 |  | nc |
| c109 | *sh4* | 1055 | A | A/T | 50854 | A | 94.2010 | 47905 | T | 5.5492 | C | 2822 |  | nc |
| m109 | *sh4* | 1055 | A | A/T | 43393 | A | 92.6232 | 40192 | T | 7.1717 | C | 3112 |  | nc |
| m754 | *sh4* | 1064 | T | T/C | 12,769 | T | 99.3730 | 12,689 | C | 0.5330 | R | 68 |  | nc |
| m754 | *sh4* | 1067 | A | A/G | 13,286 | A | 99.3530 | 13,200 | G | 0.6250 | R | 83 |  | nc |
| c109 | *sh4* | 1067 | A | A/G | 58717 | A | 95.9586 | 56344 | G | 4.0244 | C | 2363 |  | nc |
| m109 | *sh4* | 1067 | A | A/G | 43252 | A | 95.3343 | 41234 | G | 4.6495 | C | 2011 |  | nc |
| c109 | *sh4* | 1068 | T | T/G | 59527 | T | 96.0640 | 57184 | G | 3.8252 | C | 2277 |  | nc |
| m109 | *sh4* | 1068 | T | T/G | 43529 | T | 95.3801 | 41518 | G | 4.4959 | C | 1957 |  | nc |
| m754 | *sh4* | 1088 | G | G/C | 21,108 | G | 99.3750 | 20,976 | C | 0.5970 | R | 126 |  | nc |
| m754 | *sh4* | 1129 | A | A/G | 24,699 | A | 99.1340 | 24,485 | G | 0.8460 | R | 209 |  | nc |
| m754 | *sh4* | 1151 | G | G/T | 16,992 | G | 99.2530 | 16,865 | T | 0.7240 | R | 123 |  | nc |
| m109 | *sh4* | 1151 | G | G/T | 50971 | G | 98.2657 | 50087 | T | 1.6931 | R | 863 |  | nc |
| c109 | *sh4* | 1151 | G | G/T | 29193 | G | 96.5197 | 28177 | T | 3.4529 | C | 1008 |  | nc |
| m754 | *sh4* | 1171 | C | C/T | 8,240 | C | 99.0170 | 8,159 | T | 0.9830 | R | 81 |  | nc |
| m109 | *sh4* | 1171 | C | C/T | 32171 | C | 98.0573 | 31546 | T | 1.9303 | R | 621 |  | nc |
| c109 | *sh4* | 1171 | C | C/T | 18631 | C | 95.5397 | 17800 | T | 4.4227 | C | 824 |  | nc |
| m754 | *sh4* | 1175 | A | A/G | 6,762 | A | 99.3200 | 6,716 | G | 0.6360 | R | 43 |  | nc |
| m109 | *sh4* | 1175 | A | A/G | 26577 | A | 98.7207 | 26237 | G | 1.2567 | R | 334 |  | nc |
| c109 | *sh4* | 1175 | A | A/G | 16086 | A | 97.5444 | 15691 | G | 2.4245 | R | 390 |  | nc |
| m754 | *sh4* | 1187 | A | A/G | 4,409 | A | 99.3200 | 4,379 | G | 0.6800 | R | 30 |  | nc |
| m109 | *sh4* | 1187 | A | A/G | 18554 | A | 99.0299 | 18374 | G | 0.9324 | R | 173 |  | nc |
| c109 | *sh4* | 1187 | A | A/G | 11004 | A | 98.9731 | 10891 | G | 0.9724 | R | 107 |  | nc |
| m109 | *sh4* | 1200 | G | G/T | 11897 | G | 99.4116 | 11827 | T | 0.5211 | R | 62 | Glu192Stp | premature stop |
| c109 | *sh4* | 1201 | A | A/C | 6345 | A | 99.3853 | 6306 | C | 0.5201 | R | 33 | Glu192Ala | ns |
| m109 | *sh4* | 1201 | A | A/C | 9886 | A | 99.2009 | 9807 | C | 0.5867 | R | 58 | Glu192Alal | ns |
| c109 | *sh4* | 1203 | T | T/G | 5978 | T | 99.2807 | 5935 | G | 0.6189 | R | 37 | Ser193Ala | ns |
| m109 | *sh4* | 1203 | T | T/G | 9385 | T | 99.2435 | 9314 | G | 0.6713 | R | 63 | Ser193Ala | ns |
| c109 | *sh4* | 1204 | C | C/A | 5836 | C | 99.3146 | 5796 | A | 0.5655 | R | 33 | Ser193Stp | premature stop |
| m109 | *sh4* | 1204 | C | C/A | 9132 | C | 99.2006 | 9059 | A | 0.7227 | R | 66 | Ser193Stp | premature stop |
| m754 | *sh4* | 1217 | C | C/T | 1,447 | C | 99.1710 | 1,435 | T | 0.8290 | R | 12 |  | nc |
| m109 | *sh4* | 1217 | C | C/T | 6369 | C | 97.9275 | 6237 | T | 1.9783 | R | 126 | Asp197Asp | s |
| c109 | *sh4* | 1217 | C | C/T | 4497 | C | 97.8875 | 4402 | T | 2.0236 | R | 91 | Asp197Asp | s |
| m754 | *sh4* | 1232 | G | G/C | 992 | G | 98.4880 | 977 | C | 1.5120 | R | 15 |  | nc |
| c109 | *sh4* | 1232 | G | G/C | 2984 | G | 96.9839 | 2894 | C | 2.9491 | C | 88 |  | nc |
| m109 | *sh4* | 1232 | G | G/C | 3951 | G | 96.9122 | 3829 | C | 3.0119 | C | 119 |  | nc |
| m754 | *sh4* | 1243 | C | C/T | 793 | C | 98.6130 | 782 | T | 1.3870 | R | 11 | Ala206Val | s |
| c109 | *sh4* | 1243 | C | C/T | 2141 | C | 96.8706 | 2074 | T | 3.1294 | C | 67 | Ala206Val | s |
| m109 | *sh4* | 1243 | C | C/T | 2649 | C | 95.8852 | 2540 | T | 4.0770 | C | 108 | Ala206Val | s |
| m754 | *sh4* | 1262 | C | C/T | 443 | C | 97.9680 | 434 | T | 2.0320 | R | 9 |  | nc |
| c109 | *sh4* | 1262 | C | C/T | 1457 | C | 96.0879 | 1400 | T | 3.9121 | C | 57 |  | nc |
| m109 | *sh4* | 1262 | C | C/T | 1730 | C | 96.0116 | 1661 | T | 3.8728 | C | 67 | Asn212Asn | s |
| m754 | *sh4* | 1266 | C | C/A | 455 | C | 96.2640 | 438 | A | 3.7360 | C | 17 | Leu214Met | s |
| c109 | *sh4* | 1268 | G | G/A | 4279 | G | 99.1353 | 4242 | A | 0.8413 | R | 36 |  | nc |
| m754 | *sh4* | 1268 | G | G/A | 789 | G | 67.4270 | 532 | A | 32.5730 | C | 257 |  | nc |
| m754 | *sh4* | 1306 | G | G/A | 12,587 | G | 96.9890 | 12,208 | A | 2.9950 | C | 377 | Arg227His | s |
| m109 | *sh4* | 1306 | G | G/A | 36752 | G | 96.1091 | 35322 | A | 3.8529 | C | 1416 | Arg227His | s |
| c109 | *sh4* | 1306 | G | G/A | 32429 | G | 95.7045 | 31036 | A | 4.2770 | C | 1387 | Arg227His | s |
| c109 | *sh4* | 1315 | T | T/C | 40232 | T | 99.4383 | 40006 | C | 0.5269 | R | 212 | Val230Ala | s |
| m109 | *sh4* | 1315 | T | T/C | 45197 | T | 99.2500 | 44858 | C | 0.7257 | R | 328 | Val230Ala | s |
| m754 | *sh4* | 1315 | T | T/C | 27,238 | T | 90.7190 | 24,710 | C | 9.0350 | C | 2461 | Val230Ala | s |
| m754 | *sh4* | 1318 | C | C/G | 23,424 | C | 96.7600 | 22,665 | G | 0.7560 | R | 177 | Ala231Glu | ns |
| m754 | *sh4* | 1318 | C | C/T | 23,424 | C | 96.7600 | 22,665 | T | 2.3220 | R | 544 | Ala231Val | s |
| m754 | *waxy* | 84 | C | C/T | 1,624 | C | 71.6130 | 1,163 | T | 28.3870 | C | 461 |  | 5' UTR |
| c109 | *waxy* | 98 | T | T/C | 2985 | T | 99.4640 | 2969 | C | 0.5025 | R | 15 | Leu4Pro | ns |
| m754 | *waxy* | 108 | C | C/T | 2,311 | C | 87.7970 | 2,029 | T | 12.2030 | C | 282 |  | nc |
| m754 | *waxy* | 123 | G | G/A | 2,590 | G | 85.5980 | 2,217 | A | 14.4020 | C | 373 |  | nc |
| m754 | *waxy* | 153 | G | G/A | 3,124 | G | 81.5940 | 2,549 | A | 18.3420 | C | 573 |  | nc |
| c109 | *waxy* | 470 | A | A/G | 35655 | A | 98.2667 | 35037 | G | 1.5930 | R | 568 |  | nc |
| m754 | *waxy* | 655 | G | G/T | 46,028 | G | 89.4060 | 41,152 | T | 10.4980 | C | 4832 |  | nc |
| m754 | *waxy* | 688 | T | T/C | 38,644 | T | 89.0230 | 34,402 | C | 10.9230 | C | 4221 | Asp137Asp | s |
| c109 | *waxy* | 870 | C | T/C | 71475 | T | 97.8678 | 69951 | C | 2.0343 | R | 1454 |  | nc |
| m754 | *waxy* | 965 | C | A/G | 40,245 | A | 87.9070 | 35,378 | G | 11.8230 | C | 4758 |  | nc |
| c109 | *waxy* | 965 | C | A/C | 81176 | A | 97.8035 | 79393 | C | 1.7567 | R | 1426 |  | nc |
| c109 | *waxy* | 1042 | T | T/C | 87119 | T | 98.2897 | 85629 | C | 1.6632 | R | 1449 |  | nc |
| m754 | *waxy* | 1048 | A | A/T | 42,656 | A | 89.4340 | 38,149 | T | 10.2940 | C | 4391 |  | nc |
| c109 | *waxy* | 1120 | G | A/G | 81181 | A | 97.9342 | 79504 | G | 1.9857 | R | 1612 |  | nc |
| m754 | *waxy* | 1180 | C | C/A | 42,157 | C | 82.9090 | 34,952 | A | 16.9750 | C | 7156 |  | nc |
| m109 | *waxy* | 1180 | C | C/A | 46645 | C | 83.7367 | 39059 | A | 16.1625 | C | 7539 |  | nc |
| c109 | *waxy* | 1180 | C | C/A | 83695 | C | 74.0845 | 62005 | A | 25.7829 | C | 21579 |  | nc |
| m109 | *waxy* | 1254 | T | A/T | 33396 | A | 81.4349 | 27196 | T | 18.3226 | C | 6119 | Phe222Tyr | s |
| m754 | *waxy* | 1254 | T | A/T | 34,754 | A | 67.6730 | 23,519 | T | 32.1140 | C | 11161 | Phe222Tyr | s |
| c109 | *waxy* | 1254 | T | A/T | 66755 | A | 68.8952 | 45991 | T | 30.9056 | C | 20631 | Phe222Tyr | s |
| c109 | *waxy* | 1366 | C | C/T | 107513 | C | 98.5583 | 105963 | T | 1.3980 | R | 1503 |  | nc |
| m754 | *waxy* | 1374 | G | G/T | 47,806 | G | 98.7680 | 47,217 | T | 1.1610 | R | 555 |  | nc |
| m754 | *waxy* | 1393 | A | A/T | 43,219 | A | 88.3890 | 38,201 | T | 11.3650 | C | 4912 |  | nc |
| m754 | *waxy* | 1428 | C | C/A | 40,980 | C | 88.6730 | 36,338 | A | 11.2350 | C | 4604 |  | nc |
| c109 | *waxy* | 1556 | A | T/A | 75453 | T | 97.9484 | 73905 | A | 1.7759 | R | 1340 |  | nc |
| m754 | *waxy* | 1556 | A | T/A | 32,490 | T | 86.9410 | 28,247 | A | 12.8070 | C | 4161 |  | nc |
| m754 | *waxy* | 1582 | C | C/T | 36,261 | C | 89.1980 | 32,344 | T | 10.7610 | C | 3902 |  | nc |
| m754 | *waxy* | 1694 | G | G/A | 27,213 | G | 89.4540 | 24,343 | A | 10.5170 | C | 2862 | Lys309Lys | s |
| m754 | *waxy* | 1882 | C | C/A | 22,933 | C | 88.6230 | 20,324 | A | 11.2550 | C | 2581 |  | nc |
| m754 | *waxy* | 1894 | A | A/C | 24,802 | A | 87.9400 | 21,811 | C | 11.7890 | C | 2924 |  | nc |
| m109 | *waxy* | 1934 | G | A/G | 27328 | A | 81.2390 | 22201 | G | 18.7244 | C | 5117 |  | nc |
| c109 | *waxy* | 1934 | G | A/G | 51379 | A | 69.8048 | 35865 | G | 30.1777 | C | 15505 |  | nc |
| m754 | *waxy* | 1934 | G | A/G | 24,951 | A | 68.2820 | 17,037 | G | 31.6780 | C | 7904 |  | nc |
| m754 | *waxy* | 1948 | G | T/G | 22,948 | T | 86.8620 | 19,933 | G | 12.9860 | C | 2980 |  | nc |
| c109 | *waxy* | 1948 | G | T/G | 49636 | T | 98.3278 | 48806 | G | 1.4687 | R | 729 |  | nc |
| c109 | *waxy* | 2063 | G | A/G | 17640 | A | 97.8231 | 17256 | G | 2.1032 | R | 371 | Glu402Glu | s |
| m754 | *waxy* | 2063 | G | A/G | 9,309 | A | 86.1530 | 8,020 | G | 13.7070 | C | 1276 | Glu402Glu | s |
| m754 | *waxy* | 2191 | C | A/C | 22,117 | A | 86.3320 | 19,094 | C | 13.2610 | C | 2933 |  | nc |
| c109 | *waxy* | 2191 | C | A/C | 47574 | A | 98.0977 | 46669 | C | 1.4987 | R | 713 |  | nc |
| m109 | *waxy* | 2193 | G | A/G | 23641 | A | 99.4036 | 23500 | G | 0.5922 | R | 140 |  | nc |
| c109 | *waxy* | 2193 | G | A/G | 47993 | A | 97.9205 | 46995 | G | 2.0378 | R | 978 |  | nc |
| m754 | *waxy* | 2193 | G | A/G | 22,068 | A | 99.4430 | 21,945 | G | 0.5210 | R | 115 |  | nc |
| c109 | *waxy* | 2199 | C | A/C | 48744 | A | 98.5188 | 48022 | C | 1.0688 | R | 521 |  | nc |
| c109 | *waxy* | 2332 | A | G/A | 32624 | G | 98.4275 | 32111 | A | 1.5663 | R | 511 | Lys454Lys | s |
| m754 | *waxy* | 2389 | A | A/G | 12,598 | A | 95.0390 | 11,973 | G | 4.9210 | C | 620 |  | nc |
| m754 | *waxy* | 2398 | C | C/T | 15,266 | C | 90.5150 | 13,818 | T | 9.4460 | C | 1442 | Phe476Phe | s |
| m754 | *waxy* | 2469 | A | A/T | 32,814 | A | 89.8460 | 29,482 | T | 10.1180 | C | 3320 |  | nc |
| c109 | *waxy* | 2469 | A | A/T | 60923 | A | 91.6255 | 55821 | T | 8.3351 | C | 5078 |  | nc |
| m109 | *waxy* | 2469 | A | A/T | 29143 | A | 90.6255 | 26411 | T | 9.3436 | C | 2723 |  | nc |
| c109 | *waxy* | 2514 | G | C/G | 58139 | C | 98.1819 | 57082 | G | 1.7303 | R | 1006 |  | nc |
| c109 | *waxy* | 2634 | C | G/C | 43383 | G | 98.3865 | 42683 | C | 1.5029 | R | 652 |  | nc |
| m754 | *waxy* | 2634 | C | G/C | 25,187 | G | 86.7230 | 21,843 | C | 13.1850 | C | 3321 |  | nc |
| m754 | *waxy* | 2653 | A | A/G | 29,614 | A | 89.8050 | 26,595 | G | 10.1170 | C | 2996 |  | nc |
| m754 | *waxy* | 2700 | C | C/T | 36,628 | C | 88.8580 | 32,547 | T | 11.1060 | C | 4068 |  | nc |
| m754 | *waxy* | 2724 | G | G/A | 34,835 | G | 89.6000 | 31,212 | A | 10.3860 | C | 3618 |  | nc |
| c109 | *waxy* | 2729 | C | T/C | 61069 | T | 97.9253 | 59802 | C | 1.9388 | R | 1184 |  | nc |
| m109 | *waxy* | 2742 | C | T/C | 27691 | T | 99.4150 | 27529 | C | 0.5200 | R | 144 |  | nc |
| m754 | *waxy* | 2742 | C | T/C | 28,584 | T | 99.2900 | 28,381 | C | 0.6370 | R | 182 |  | nc |
| c109 | *waxy* | 2742 | C | T/C | 58403 | T | 97.7998 | 57118 | C | 2.1626 | R | 1263 |  | nc |
| m754 | *waxy* | 2794 | C | C/G | 22,270 | C | 83.4260 | 18,579 | G | 16.5020 | C | 3675 | Thr534Ser | s |
| c109 | *waxy* | 2794 | C | C/G | 44985 | C | 72.1863 | 32473 | G | 27.7026 | C | 12462 | Thr534Ser | s |
| m109 | *waxy* | 2794 | C | C/G | 25493 | C | 83.6779 | 21332 | G | 16.2084 | C | 4132 | Thr534Ser | s |
| m754 | *waxy* | 2890 | A | A/G | 26,019 | A | 88.9350 | 23,140 | G | 10.9880 | C | 2859 |  | nc |
| m754 | *waxy* | 3054 | A | A/G | 17,494 | A | 90.5970 | 15,849 | G | 9.3400 | C | 1634 | Lys582Glu | ns |
| m754 | *waxy* | 3128 | G | G/T | 4,586 | G | 89.7300 | 4,115 | T | 10.2050 | C | 468 |  | 3' UTR |
| m109 | *waxy* | 3162 | A | A/G | 5437 | A | 99.4298 | 5406 | G | 0.5150 | R | 28 |  | 3' UTR |
| m754 | *waxy* | 3182 | A | A/C | 9,943 | A | 90.6170 | 9,010 | C | 9.1620 | C | 911 |  | 3' UTR |
| m109 | *waxy* | 3221 | G | G/A | 14213 | G | 98.4451 | 13992 | A | 1.2453 | R | 177 |  | 3' UTR |
| m109 | *waxy* | 3224 | G | G/A | 20775 | G | 99.1528 | 20599 | A | 0.5969 | R | 124 |  | 3' UTR |
|  |  |  |  |  |  |  |  |  |  |  |  |  |  |  |
| Putative EMS mutation | | |  |  |  |  |  |  |  |  |  |  |  |  |
| Putative premature stop codon | | |  |  |  |  |  |  |  |  |  |  |  |  |
| SNP in wild population of *M. stipoides (Fitzgerald et al, 2011)* | | | | |  |  |  |  |  |  |  |  |  |  |
